# A multi-view graph convolutional network framework based on adaptive adjacency matrix and multi-strategy fusion mechanism for identifying spatial domains

**DOI:** 10.1093/bioinformatics/btaf172

**Published:** 2025-04-15

**Authors:** Yuhan Fu, Mengdi Nan, Qing Ren, Xiang Chen, Jie Gao

**Affiliations:** School of Science, Jiangnan University, Wuxi, Jiangsu 214122, China; School of Science, Jiangnan University, Wuxi, Jiangsu 214122, China; School of Science, Jiangnan University, Wuxi, Jiangsu 214122, China; School of Science, Jiangnan University, Wuxi, Jiangsu 214122, China; School of Science, Jiangnan University, Wuxi, Jiangsu 214122, China

## Abstract

**Motivation:**

Spatial transcriptomics (ST) addresses the loss of spatial context in single-cell RNA-sequencing by simultaneously capturing gene expression and spatial location information. A critical task of ST is the identification of spatial domains. However, challenges such as high noise levels and data sparsity make the identification process more difficult.

**Results:**

To tackle these challenges, STMGAMF, a multi-view graph convolutional network model that employs an adaptive adjacency matrix and a multi-strategy fusion mechanism is proposed. STMGAMF dynamically adjusts the edge weights to capture complex spatial structures during training by implementing the adaptive adjacency matrix and optimizes the embedded features through the multi-strategy fusion mechanism. STMGAMF is evaluated on multiple ST datasets and outperforms existing algorithms in tasks like spatial domain identification, visualization, and spatial trajectory inference. Its robust performance in spatial domain identification and strong generalization capability position STMGAMF as a valuable tool for unraveling the complexity of tissue structures and underlying biological processes.

**Availability and implementation:**

Source code is available at Github (https://github.com/Fuyh0628/STMGAMF) and Zenodo (https://zenodo.org/records/15103358).

## 1 Introduction

The spatial distribution of cells within tissues is important for understanding their biological functions and their expression in pathological states (Longo *et al.* 2021). Spatial transcriptomics (ST), as a revolutionary and cutting-edge tool, is capable of capturing both gene expression and spatial location information in a tissue context. This technology breaks through the limitations of traditional transcriptomics technology on tissue context and spatial resolution and has achieved remarkable results in the study of various biological systems. Spatial domain identification is one of the core tasks in analyzing ST data, which identifies and delineates functional regions with similar gene expression characteristics and spatial consistency by analyzing the spatial patterns of gene expression in tissues. Through spatial domain identification, the spatial distribution patterns of different cell types can be analyzed, the microenvironmental relationship network at the cellular level can be constructed, the interactions between cells can be revealed in-depth, and the mechanism of multicellular collaboration can be analyzed, which provides important clues for the study of cellular heterogeneity and tissue dynamics in complex diseases such as tumors (Galeano Niño *et al.* 2022).

The spatial domain identification task faces three significant challenges: high noise, data sparsity, and resolution limitation. In recent years, researchers have proposed various algorithms to improve the accuracy and stability of spatial domain identification. High noise in ST data mainly comes from low-throughput signal dilution, spatial coding impairments, and background noise generated by sequencing errors. SEDR combined variational graph autoencoder and deep masked autoencoder network to optimize the low-dimensional representation and enhance noise immunity (Xu *et al.* 2024). spaVAE was based on a negative binomial model-based decoder to minimize the effect of noise (Tian *et al.* 2024). sGAE innovatively transformed single-cell gene expression matrices into stable gene association network entropy matrices and enhanced data stability through dynamic network entropy analysis (Zhong *et al.* 2023). IRIS integrated single-cell RNA sequencing data to infer cell-type composition to minimize the impact of technical noise and improve bio-interpretability (Ma and Zhou 2024). Data sparsity is another key factor limiting ST data analysis. Insufficient sequencing depth or low gene expression in a specific region can lead to information loss and affect the integrity of the null domain. STAGATE learned spatial neighbor relationships through the graph attention mechanism to improve feature learning in low-expression regions (Dong and Zhang 2022). Spatial-MGCN (Wang *et al.* 2023) and SpaNCMG (Si *et al.* 2024) utilized a multi-view graph convolutional network (GCN) to integrate gene expression and spatial structural information, enabling neighborhood information to enhance sparse data. MuCoST (Zhang *et al.* 2024) and stCluster (Wang *et al.* 2024a) combined contrastive learning (CL) to optimize feature extraction and learning for low-expression data. In addition, different ST platforms (e.g. 10×Visium, Slide-seq, MERFISH, and Stereo-seq) have different spatial resolutions, which affects the finesse of spatial domain identification. GAAEST improved the ability to identify spatial domains at cellular resolution through the synergistic optimization of graph attention network and self-supervised learning (Wang *et al.* 2024b). DeepST introduced the domain adversarial neural network to effectively solve the cross-platform resolution difference problem (Xu *et al.* 2022). GraphST used self-supervised learning and contrast loss strategy within a graph neural network framework to improve resolution adaptation (Long *et al.* 2023). CytoCommunity proposed a differentiable graph pooling technique, which effectively solves the problem of cross-sample graph alignment through a supervised learning framework (Hu *et al.* 2024). SpatialGlue improved spatial domain identification by integrating multiple spatial histology data from the same tissue and slices (Long *et al.* 2024). Although these algorithms have made significant progress in dealing with high noise, data sparsity, and resolution limitations, they still suffer from generally high computational complexity, sensitivity to data quality, and limited ability to generalize across datasets. Therefore, developing practical algorithms to adapt to complex data characteristics remains a central research goal of ST.

We propose STMGAMF, a multi-view graph convolutional network model that employs an adaptive adjacency matrix and a multi-strategy fusion mechanism. STMGAMF first considers spatial and gene expression similarity comprehensively by constructing spatial and feature adjacency graphs, thus overcoming the problem that a single adjacency cannot adequately capture the interactions between gene expression data and spatial information. Second, STMGAMF introduces an adaptive adjacency matrix to optimize the graph structure based on the data characteristics, which overcomes the limitations of static, predefined adjacencies in traditional algorithms when dealing with complex spatial structures, thus better adapting to the complexity of the data. Subsequently, in designing the multi-view graph convolutional network encoder, STMGAMF introduces adaptive adjacency matrices in the spatial convolution and feature convolution modules, respectively. In contrast, the joint convolution module uses the original adjacency matrix. This strategy enhances the model’s flexibility to extract spatial and feature information while preserving the global commonality information in the data. Finally, STMGAMF proposes a multi-strategy fusion mechanism that combines the adaptive ability of the attention mechanism with the stability of the fixed learnable parameters, overcoming the problem that simple splicing or fixed weighted averaging to fuse different features cannot dynamically adjust the importance of the features according to the differences in the data distribution, and improves the model’s stability and generalization ability. We evaluate STMGAMF on several downstream analysis tasks, including spatial domain identification, visualization, spatial trajectory inference, gene expression imputation, and differential expression analysis. The results indicate that STMGAMF performs competitively compared to seven other popular algorithms.

## 2 Materials and methods

### 2.1 Data preprocessing

STMGAMF takes gene expression and spatial location information from ST data as its input. To reduce bias from technical or background noise in the ST data, all datasets are initially filtered to exclude spatial areas outside the main tissue areas. The raw gene expression typically contains many low-expression or low-variation genes, and we use the SCANPY toolkit to genetically filter and select features from the data, identifying the top 3000 highly variation genes. Finally, they are normalized using a scale factor. The normalization function is:

(1) $${\text{expression}}_{ij}=\frac{{\text{count}}_{ij}}{\sum_{j}{\text{count}}_{ij}}\times 10000.$$

### 2.2 Graph construction

To integrate gene expression and spatial location information effectively, we employ various adjacency graph construction methods based on spatial proximity and gene expression similarity. First, spatial proximity is assessed by analyzing the locations of adjacent spots within the tissue. We construct a spatial adjacency graph $G_{s}=\left(A_{s},X\right)$, where $A_{s}\in R^{N\times N}$ is the spatial adjacency matrix of N spots and $X\in R^{N\times M}$ denotes the normalized gene expression matrix, where M is the number of filtered genes. If both spot i and j are within a predefined radius r, then we set $A_{ij}^{s}=A_{ji}^{s}=1$, and 0 otherwise (for the DLPFC, r=560, while for the other datasets r=15). The spatial adjacency graph accurately represents the tissue’s spatial structure and ensures that the model reflects direct proximity based on physical location within the tissue. The graph takes into account only the spatial proximity of cells, but it does not effectively represent the biological significance of variations in gene expression.

To overcome the limitations of the spatial adjacency graph, we construct a feature adjacency graph based on gene expression similarity. This graph identifies cellular areas within a tissue that have similar functions or states by assessing the similarity of gene expression vectors, even if these areas are physically far apart. Specifically, we construct the k-nearest neighbor feature adjacency graph $G_{f}=\left(A_{f},X\right)$, where $A_{f}\in R^{N\times N}$ is the feature adjacency matrix of N spots. The edge weights of the feature adjacency matrix are calculated based on the cosine similarity. If spot j is a neighbor of spot i, then we set $A_{ij}^{f}=1$, and 0 otherwise (k=15 by default). The feature adjacency graph effectively identifies cells or areas with similar gene expression patterns. However, it may sometimes mistakenly group spatially distant spots in adjacent areas, overlooking the spatial structure. By building both a spatial adjacency graph and a feature adjacency graph, we enable the model to leverage spatial proximity and gene expression similarity simultaneously.

### 2.3 Adaptive adjacency matrix construction

Traditional static adjacency matrices are usually created with predefined relationships. However, this approach may struggle to accurately depict the complex spatial structure of an organization or sample. In contrast, the adaptive adjacency matrix is designed to optimize relationships between spots by dynamically adjusting to changes in the input data. Here, we construct the adaptive adjacency matrix $A^{\text{adapt}}$ using both spatial adjacency and feature adjacency between the spots. The specific form is:

(2) $$A^{{}^{\text{adapt}}}=\text{SparseMatrix}\left(\text{indices},\text{values}\right),$$

where *indices* is the position index of non-zero elements in the adjacency matrix, indicating which spots are connected to each other; *values* is the weight of edges in the adjacency matrix.

### 2.4 Multi-view graph convolutional network encoder

In this model, we develop a multi-view learning framework based on a GCN. The goal is to fully integrate the spatial and feature information within the graph structure while capturing the dependencies and interrelationships between various spots. The framework consists of three main modules: the spatial convolution module, the feature convolution module, and the joint convolution module.

#### 2.4.1 Spatial convolution and feature convolution

The purpose of the spatial convolution module is to capture the spatial information of the graph structure. Performing convolution operations on the spatial adaptive adjacency matrix $A_{s}^{{}^{\text{adapt}}}$ allows for identifying and capturing spatial correlations between spots. The feature convolution module is similar to the spatial convolution module, where we perform the convolution operation on the feature adaptive adjacency matrix $A_{f}^{{}^{\text{adapt}}}$. The multilayer convolution network applies the following hierarchical propagation rules:

(3) $$H^{\left(l+1\right)}=\text{ReLU}\left(A^{{}^{\text{adapt}}}{\tilde{D}}^{-\frac{1}{2}}\tilde{A}{\tilde{D}}^{-\frac{1}{2}}H^{\left(l\right)}W^{\left(l\right)}\right),$$

where $W^{\left(l\right)}$ is the weight matrix of the l-th layer in the spatial convolution and the feature convolution; the initial $H^{\left(0\right)}=X$; ReLU is the activation function; $\tilde{A}=A+I$, and $\tilde{D}$ is the diagonal degree matrix of $\tilde{A}$, with the diagonal element ${\tilde{D}}_{ii}=\sum_{j=1}^{N}{\tilde{A}}_{ij}$.

#### 2.4.2 Joint convolution

In graph data, there is often a potential correlation between spatial information and feature information. In order to fully utilize the common information between the two, we design a joint convolution module. The module performs convolution operations on the spatial adjacency matrix $A_{s}$ and feature adjacency matrix $A_{f}$ respectively, to extract the common information. The propagation rules are defined as follows:

(4) $$H_{j}^{\left(l+1\right)}=\text{ReLU}\left({\tilde{D}}^{-\frac{1}{2}}\tilde{A}{\tilde{D}}^{-\frac{1}{2}}H_{j}^{\left(l\right)}W_{j}^{\left(l\right)}\right),$$

where $W_{j}^{\left(l\right)}$ is the weight matrix of the l-th layer in the joint convolution, and the initial $H_{j}^{\left(0\right)}=X$. Joint convolution shares parameters between the spatial graph and the feature graph.

By using joint convolution, we can obtain the spatial joint embedding $H_{sj}$ and feature joint embedding $H_{fj}$, which can further define the joint embedding $H_{j}$ as:

(5) $$H_{j}=\frac{H_{sj}+H_{fj}}{2}.$$

To improve the consistency of the representation between $H_{sj}$ and $H_{fj}$, we introduce consistency constraints defined as follows:

(6) $$L_{\text{con}}={\left\|{\tilde{H}}_{sj}{\tilde{H}}_{sj}^{T}-{\tilde{H}}_{fj}{\tilde{H}}_{fj}^{T}\right\|}_{2}^{2},$$

where ${\tilde{H}}_{sj}$ and ${\tilde{H}}_{fj}$ are the normalization matrices of $H_{sj}$ and $H_{fj}$, respectively. This constraint promotes consistency between spatial embedding and feature embedding, enhancing the model’s embedding quality.

### 2.5 Multi-strategy fusion

To enhance identification accuracy, we design a multi-strategy fusion mechanism that adaptively integrates these features. The mechanism combines an attention-based fusion method and a fixed learnable parameter-based fusion method.

#### 2.5.1 Fusion based on the attention mechanism

The attention mechanism highlights essential information while reducing irrelevant features by adjusting input feature weights adaptively, effectively capturing global dependencies. The specific process is as follows:

(7) $$\left(a_{j},a_{s},a_{f}\right)=\text{att}\left(H_{j},H_{s},H_{f}\right),$$

where $a_{j}$, $a_{s}$, and $a_{f}$ are the attention coefficients of $H_{j}$, $H_{s}$, and $H_{f}$, respectively. In this approach, all three embeddings use the same shared vector to compute the attention coefficients. After calculating the attention coefficients for each embedding, we combine them, weighting each one according to its respective attention coefficient, to obtain the final attention fusion embedding $H_{\text{attention}}$:

(8) $$H_{\text{attention}}=F\left(a_{j}\cdot H_{j}+a_{s}\cdot H_{s}+a_{f}\cdot H_{f}\right),$$

where F is a single linear layer used to learn highly variable features of the underlying representation.

#### 2.5.2 Fusion based on fixed learnable weight parameters

Fixed learnable weight parameter-based fusion is a strategy for combining features through weighted summation and is particularly effective at capturing the overall relationships among different embedded features. In contrast to the attention mechanism, this approach emphasizes the stability of the fusion process while incorporating learnable weight parameters to adjust the weights of the embeddings dynamically. We introduce three learnable parameters, denoted $c_{j}$, $c_{s}$, and $c_{f}$, to dynamically adjust the weights of different embeddings. These parameters allow us to capture the interrelationships among $H_{j}$, $H_{s}$, and $H_{f}$ to obtain a weighted combination of features. The details are as follows:

(9) $$H_{\text{learnable}}=\tanh\left(c_{j}\right)\cdot H_{j}+\tanh\left(c_{s}\right)\cdot H_{s}+\tanh\left(c_{f}\right)\cdot H_{f}.$$

#### 2.5.3 Final embedding

After computing both embeddings $H_{\text{attention}}$ and $H_{\text{learnable}}$, we fuse these two representations by averaging them to obtain the final embedding representation H:

(10) $$H=\frac{H_{\text{attention}}+H_{\text{learnable}}}{2}.$$

This fusion merges the adaptive capability of the attention mechanism with the stability of fixed learnable weight parameters, enabling flexibility in capturing the significance of different embeddings while maintaining representation stability.

### 2.6 Zero-inflated negative binomial decoder

In order to address the issues of zero inflation and missing data in ST data, the Zero-Inflated Negative Binomial (ZINB) decoder structure is employed. This structure helps reconstruct the gene expression matrix and captures the complex global information inherent in ST data. ZINB is defined as follows:

(11) $$f_{\text{ZINB}}\left(x|\pi ,\mu ,\theta \right)=\pi \delta_{0}\left(x\right)+\left(1-\pi \right)f_{\text{NB}}\left(x|\mu ,\theta \right)$$

 

(12) $$f_{\text{NB}}\left(x|\mu ,\theta \right)=\frac{\Gamma \left(x+\theta \right)}{\Gamma \left(x+1\right)\Gamma \left(\theta \right)}{\left(\frac{\theta }{\theta +\mu }\right)}^{\theta }{\left(\frac{\mu }{\theta +\mu }\right)}^{x},$$

where μ denotes the mean; θ denotes the dispersion; π denotes the probability of a zero value; $\delta_{0}$ denotes an indicator function that $\delta_{0}=1$ when x=0 and 0 otherwise.

The negative log-likelihood of ZINB serves as the reconstruction loss for the original gene expression, which can be formulated as:

(13) $$\begin{array}{c} L_{\text{ZINB}}=-\sum_{i=1}^{N}(\delta_{0}(x_{i})\;\text{log}\;\left(\pi_{i}+(1-\pi_{i}){\left(\frac{\theta_{i}}{\theta_{i}+\mu_{i}}\right)}^{\theta_{i}}\right)\\ +(1-\delta_{0}(x_{i}))\left(\text{log}(1-\pi_{i})+\text{log}\;f_{\text{NB}}(x_{i}|\mu_{i},\theta_{i}))\right)\\ +\lambda \sum_{i=1}^{N}\pi_{i}^{2}, \end{array}$$

where the regularization term $\lambda \sum_{i=1}^{N}\pi_{i}^{2}$ is used to control the size of the zero-value probability parameter π, which enhances the stability and generalization of the model.

### 2.7 Spatial regularization constraints

The spatial locations and gene expression patterns of cells or spatial spots within an organizational structure typically exhibit significant spatial dependence. Adjacent cells or spatial spots tend to show similar gene expression characteristics, suggesting they may belong to the same functional area. In contrast, more distant spots exhibit greater gene expression differences and may correspond to different areas (Stoltzfus *et al.* 2020). To take full advantage of this spatial structural feature, we introduce spatial regularization constraints and use both similarity information and spatial adjacency information to calculate the regularization constraint loss. Specifically, the spatial regularization constraint is based on the learned potential embeddings H. The similarity matrix S is constructed based on the cosine similarity, which quantifies the degree of similarity between spatial spots. To enhance the similarity between adjacent spots and reduce the similarity between non-adjacent spots, we define the spatial regularization loss as follows:

(14) $$\begin{array}{c} L_{\text{reg}}=-\frac{1}{2}(\frac{1}{\left|G_{\text{nei}}\right|}\sum_{\left(i,j\right)\in G_{\text{nei}}}\;\text{log}\;\sigma \left(S_{ij}\right)\\ +\frac{1}{\left|G_{\text{neg}}\right|}\sum_{\left(i,j\right)\in G_{\text{neg}}}\;\text{log}\;\left(1-\sigma \left(S_{ij}\right)\right)), \end{array}$$

where $G_{\text{nei}}$ denotes the relationship between adjacent spots, and $G_{\text{neg}}$ denotes the relationship between non-adjacent spots.

By maximizing the similarity between adjacent spots and minimizing the similarity between non-adjacent spots, the model can create a spatial representation in potential space that aligns with the biological structure.

### 2.8 Model loss function

During the model training process, we jointly optimize the multi-view GCN encoder, the ZINB decoder, and the spatial regularization constraints to enhance the model’s performance.

The final loss function of the model is defined as:

(15) $$L=\alpha L_{\text{ZINB}}+\beta L_{\text{con}}+\gamma L_{\text{reg}}.$$

In this loss function, we set weight parameters α=1, β=10, and γ=0.1 to balance the effects of consistency loss, reconstruction loss, and spatial regularization loss.

Detailed methodological procedures are presented in Supplementary Section S1.

## 3 Results

### 3.1 Overview of STMGAMF

**Figure 1.**
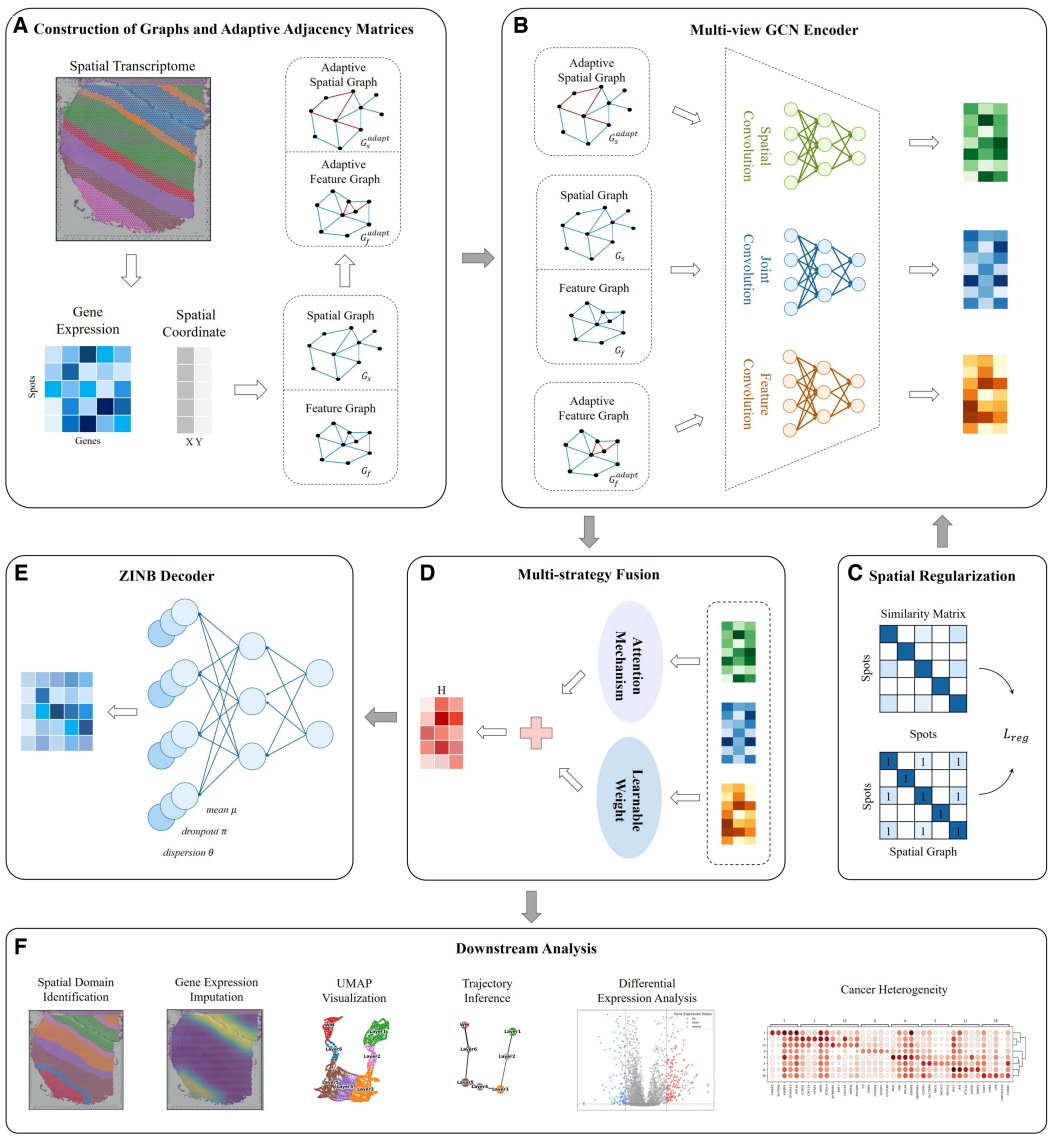
Overview of STMGAMF. (A) Construction of graphs and adaptive adjacency matrices. The spatial and feature adjacency graphs are constructed based on different criteria, and the adaptive adjacency matrix is introduced to adjust the edge weights dynamically. (B) Multi-view GCN encoder. STMGAMF integrates the spatial, feature and their common information in the graph structure using a multi-view GCN encoder. (C) Spatial regularization. STMGAMF preserves the spatial structure using spatial regularization constraints. (D) Multi-strategy Fusion. STMGAMF adaptively integrates spatial, feature and joint embeddings through a multi-strategy fusion mechanism to obtain the final embedding representation. (E) ZINB Decoder. STMGAMF reconstructs gene expression using the ZINB decoder. (F) Downstream analysis. STMGAMF clusters the learned embedding representations and implements downstream analysis tasks.

STMGAMF is a novel spatial domain identification algorithm that utilizes a multi-view GCN with an adaptive adjacency matrix and a multi-strategy fusion mechanism (Fig. 1). In order to more effectively integrate gene expression with spatial location information and fully explore adjacency information, STMGAMF constructs both a spatial adjacency graph $G_{s}$ and a feature adjacency graph $G_{f}$. To enhance the capacity of the adjacency graph to capture spatial structural complexity, STMGAMF incorporates an adaptive adjacency matrix. This matrix is built upon the spatial adjacency relationship and feature adjacency relationship among spots (Fig. 1A). During model training, the adaptive adjacency matrix dynamically adjusts the weights of the edges, enabling the model to learn the adjacency graph automatically. To extract information from specific perspectives, STMGAMF employs a multi-view GCN that performs graph convolution on each adjacency matrix separately. Furthermore, STMGAMF contains a joint convolution module that extracts the joint embedding from both the spatial graph and the feature graph using a parameter-sharing strategy, which maximizes the use of common information between the two graphs (Fig. 1B). Given that different embedding features contribute variably to the model’s overall performance, STMGAMF adaptively integrates these features using a multi-strategy fusion mechanism to achieve the final embedding representation H (Fig. 1D). In order to align with the characteristics of ST data, STMGAMF utilizes a ZINB decoder structure, which allows for the reconstruction of the gene expression matrix and captures the complexity of the data from a holistic perspective (Fig. 1E). At the same time, in order to further maintain the integrity of the spatial structure, STMGAMF introduces a spatial regularization constraint (Fig. 1C). This constraint significantly enhances the embedding’s ability to express spatial structures by strengthening the similarity among adjacent spots while suppressing similarities between non-adjacent spots. Finally, STMGAMF jointly optimizes the multi-view GCN encoder, ZINB decoder, and spatial regularization constraints, resulting in learned embedding representations that can be used for various downstream analysis tasks (Fig. 1F). We apply STMGAMF to the human dorsolateral prefrontal cortex (DLPFC), human breast cancer, anterior tissue of mouse brain datasets obtained by the 10× Visium platform, and coronal mouse olfactory bulb dataset obtained by the Stereo-seq platform, respectively, of which the results of the latter three datasets are described in detail in Supplementary Section S3.

### 3.2 STMGAMF enhances the accuracy of spatial domain identification

**Figure 2.**
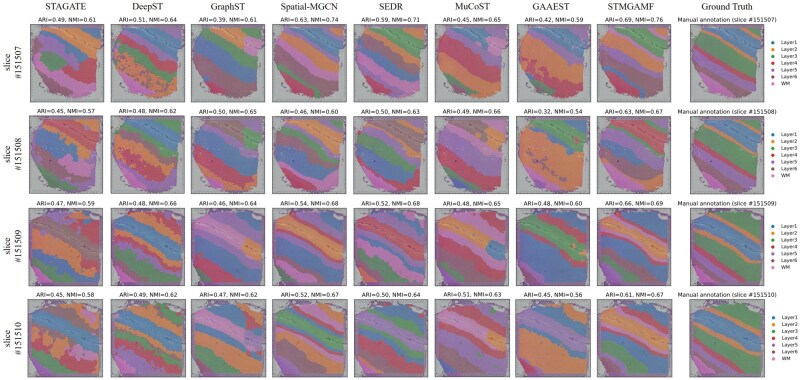
Manual annotation results and spatial domain identification results in the DLPFC for STMGAMF and the seven baseline algorithms, focusing on slices #151507-#151510.

To quantitatively assess the spatial clustering performance of STMGAMF, we select the 10× Visium dataset of the DLPFC, which includes spatial expression information from 12 slices (Maynard *et al.* 2021). This dataset is manually annotated based on morphological features and genetic markers by Maynard *et al.* (2021) who categorized the six cortical layers (L1–L6) of the DLPFC as well as the white matter (WM) (Fig. 2). These manual annotations provide a reliable reference standard for evaluating model performance. In our study, we test the accuracy of STMGAMF against seven other mainstream spatial clustering algorithms: STAGATE, DeepST, GraphST, Spatial-MGCN, SEDR, MuCoST, and GAAEST. The goal is to identify biologically meaningful spatial domains. STMGAMF efficiently identifies the expected cortical layer structure and demonstrates significant improvements compared to the other seven algorithms. A visual comparison of the spatial structural domains identified by these algorithms across tissue sections #151507-#151510 illustrates the differences. STAGATE and GAAEST only recover the clustering for cortical layer L1. DeepST struggles to distinguish between cortical layers L4–L6, failing to achieve clear separations and leading to numerous mixing spots within those areas. Although GraphST and MuCoST generate cortical layers that resemble the annotations, they inaccurately represent the thickness of these layers and mixed in other layers with the clustering for L1. None of these algorithms correctly identifies the full number of cortical layers, resulting in a division of the layers into only five categories. Spatial-MGCN, SEDR, and STMGAMF successfully map the complete structure of cortical layers L1–L6 and white matter. However, Spatial-MGCN fails to accurately distinguish between cortical layer L6 and white matter in slice #151510. At the same time, SEDR has confounding errors in identifying layers L2 and L3 in slice #151507, and the boundaries are not smooth enough. In contrast, STMGAMF is the only algorithm to identify the cortical layers L1–L6 and WM in high agreement with the manual annotations and establishes neuroscience definitions. It clearly captures the ‘sandwich’ morphology of cortical layers L1–L3 as indicated in the annotations and effectively detects the deeper L4–L6, which the other algorithms struggle to differentiate. Additionally, STMGAMF presents a relatively clear and intact representation of the white matter.

For quantitative evaluation, we assess the clustering performance of each algorithm using several metrics: Adjusted Rand Index (ARI), Normalized Mutual Information (NMI), Homogeneity Score (HS), Completeness Score (CS), and V-measure. These algorithms are applied to all slices of the DLPFC (Supplementary Figs S1 and S2). STMGAMF achieves the best performance, with the highest median ARI of 0.61 and the highest median NMI of 0.68. Spatial-MGCN and SEDR follow closely with median ARI values of 0.56 and 0.55 and median NMI values of 0.67 and 0.68, respectively. MuCoST yields a median ARI of 0.53 and a median NMI of 0.67, while GraphST achieves a median ARI of 0.51 and a median NMI of 0.66. Notably, the other three algorithms do not achieve a median ARI above 0.50. In particular, for slices #151507, STMGAMF clearly delineates the layer boundaries and achieves the highest clustering accuracies, with an ARI of 0.69 and NMI of 0.76 (Fig. 2). This outcome indicates that STMGAMF can capture the complexity of spatial structure more accurately.

Furthermore, in order to thoroughly assess the performance of STMGAMF and its capability to generalize to downstream tasks, we visualize the 2D Uniform Manifold Approximation and Projection (UMAP) space of the embeddings generated by various algorithms applied across 12 slices of DLPFC. This visualization is combined with partition-based graph abstraction (PAGA) to infer spatial trajectories (Supplementary Fig. S3). In particular, for slices #151507, the UMAP generated from STMGAMF embeddings clearly demonstrates a structured layering, with spots belonging to the same manually annotated layer clustering together (Supplementary Fig. S4A). Additionally, spots from different layers are distinctly separated, exhibiting well-defined boundaries. Both HS and CS achieve the score of 0.76, marking the best performance. This indicates that STMGAMF effectively and accurately reveals the spatial organization of the DLPFC. Furthermore, the PAGA maps illustrate a developmental pathway consistent with the temporal developmental order of the cortical layers (Supplementary Fig. S4B). This finding aligns with the functional similarities between adjacent cortical layers and their temporal progression (Gilmore and Herrup 1997). These results surpass the performance of other baseline algorithms. In the UMAP visualization results produced by GAAEST, the spots of different layers are mixed and not separated. Cortical layers L4–L6 of STAGATE are not correctly distinguished as laminae. Similarly, DeepST and MuCoST struggle to clearly separate spots from different layers, and spots from the same layer lack tight clustering. Additionally, GraphST has difficulty accurately identifying cortical layer L6 and some spots in cortical layer L4 are partially fused with those in cortical layer L5. The boundaries between the different cortical layers of the SEDR are not distinct enough and are slightly mixed. In contrast, both STMGAMF and Spatial-MGCN reflect a consistent relationship between spatial trajectories and spatial maps. However, Spatial-MGCN produces UMAP visualizations with slightly blended speckles in cortical layers L4 and L5, whereas STMGAMF presents a clearer delineation with a biologically consistent organization embedded in space. This experiment validates STMGAMF’s ability to enhance the spatial domain identification performance of ST data while preserving the underlying biological features necessary for effective representation in downstream tasks.

Additionally, the density heatmap based on UMAP further confirms the advantages of STMGAMF in the embedding space (Supplementary Fig. S4C). It is evident that the density distributions of different clusters generated by the STMGAMF model are continuous, with good intraclass compactness observed in high-density areas. This clearly demonstrates the hierarchical structure, indicating that the model can accurately reflect the internal consistency of the clusters in terms of feature representation. The heatmap also shows that the hierarchical structure of the spatial domain is preserved not only in the local density distribution but also in the clarity and separation of boundaries between different clusters. The hierarchical structure in the density heatmap aligns with the identification results of the spatial domains, demonstrating that the model preserves the topological relationship between spatial domains in the feature embedding. These results further verify that STMGAMF employs a multi-strategy fusion mechanism to ensure high embedding quality and to fully learn the representation of spatial information and inter-domain boundaries.

**Figure 3.**
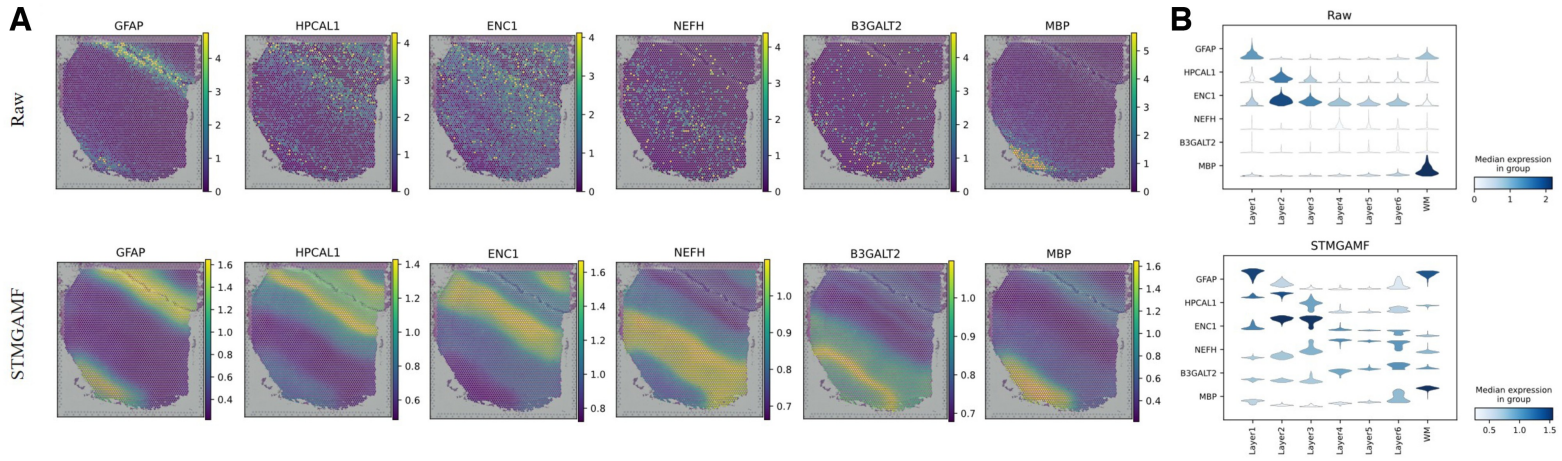
STMGAMF reconstructs the expression of layer marker genes on slice #151507 in the DLPFC. (A) Visualization of the original and reconstructed expression patterns of layer marker genes on slice #151507 in the DLPFC. (B) Violin plots of original layer marker gene expression and violin plots of STMGAMF reconstructed layer marker gene expression.

Raw ST data are often affected by high levels of noise and significant loss events, which can hinder the accuracy of gene expression analysis. To address this, robust representation learning algorithms should effectively distinguish between irrelevant confounding factors and the critical information regarding tissue distribution present in the raw data. In our study, we apply the STMGAMF to the DLPFC to uncover the spatial expression patterns of genes with greater accuracy and resolution. We select layer marker genes in each of the six layers—GFAP, HPCAL1, ENC1, NEFH, B3GALT2, and MBP—from DLPFC section #151507 and compare their raw gene expression levels with the expression reconstructed using STMGAMF (Fig. 3A). In the analysis of raw gene expression, we observe significant confounding effects impacting all layer marker genes. For instance, NEFH exhibits a spatially dispersed distribution that lacked clear pattern characteristics. However, after reconstructing the expression data, NEFH reveals high expression areas within cortical layer L5 as well as adjacent layers, clearly showcasing layer-specific expression patterns. To further validate the denoising capabilities of STMGAMF, we analyze the original expression data alongside the reconstructed expression using violin plots (Fig. 3B). The results indicate that the spatial expression patterns of these layer-tagged genes are significantly enhanced following reconstruction. Overall, our findings suggest that STMGAMF, which utilizes a ZINB decoder, is better suited for adapting to the features of ST data, effectively reducing noise and data loss while preserving significant biological information.

## 4 Conclusion

Spatial domain identification is a key challenge in ST due to high noise levels and data sparsity. To address this, we propose STMGAMF, a novel spatial domain identification algorithm. STMGAMF integrates spatial and feature adjacency graphs, considering both spatial and gene expression similarity, overcoming the limitations of relying on a single adjacency relationship. Unlike traditional methods relying on static adjacency relationships, STMGAMF introduces the adaptive adjacency matrix to optimize graph structures dynamically for complex tissue architectures. Furthermore, its multi-view GCN enhances model flexibility and robustness, while the multi-strategy fusion mechanism dynamically adjusts feature importance, improving stability and generalization.

We verify the superior performance of STMGAMF in spatial transcriptome data analysis and compare the results with seven mainstream algorithms. The results show that STMGAMF exhibits excellent spatial domain identification capabilities. In the DLPFC, the results in Fig. 2 and Supplementary Fig. S4 show that STMGAMF not only successfully distinguishes the six cortical layers (L1–L6) and WM, accurately captures the layer structure that conforms to the neuroscience definition, but also performs best in all evaluation indicators. The median ARI is 0.61, and the median NMI is 0.68. In addition to its excellent spatial domain identification performance, STMGAMF effectively removes high noise in ST data and retains key biological features. Regarding gene expression pattern reconstruction, the results in Fig. 3 show that STMGAMF significantly enhances the spatial expression specificity of layer marker genes and reveals significant patterns of genes in the organizational hierarchy. In the human breast cancer, STMGAMF verifies its performance in highly heterogeneous cancer tissues with the highest spatial domain identification accuracy. ARI improves by about 0.10–0.35, and NMI improves by about 0.07–0.13 compared with other algorithms. The results in Supplementary Figs S5 and S6 show that STMGAMF clearly distinguishes different spatial domains and enhances gene expression through denoising, revealing important cancer biological characteristics. For example, the differentially expressed gene analysis in DCIS/LCIS and IDC regions provides new clues for cancer mechanism research and clinical diagnosis, demonstrating the practical application potential of STMGAMF. In the mouse anterior brain, the results in Supplementary Fig. S7 show that STMGAMF exhibits a clear hierarchical structure and natural smooth boundaries, accurately depicting the spatial heterogeneity of tissues, achieving an optimal ARI of 0.53 and a suboptimal NMI of 0.71. In addition, in the coronal mouse olfactory bulb obtained by the high-resolution Stereo-seq platform, the results in Supplementary Fig. S8 show that STMGAMF further demonstrates its adaptability to complex spatial structures. Compared with the limitations of other algorithms in hierarchical structure identification, STMGAMF can accurately parse the anatomical domain of the olfactory bulb, successfully distinguish the inner and outer tissues, and highly match the known tissue annotations. These results suggest that STMGAMF can be used to identify ST organizational structures at different spatial resolutions. We also perform ablation experiments on DLPFC and human breast cancer, and the results are shown in Supplementary Section S5, where STMGAMF achieves the best performance among all variants.

In conclusion, STMGAMF not only demonstrates excellent advantages in analyzing complex biological data but also promotes an in-depth understanding of life micro-mechanisms by its ability to accurately reveal the spatial structure and functional properties of biological tissues, providing powerful support for life science research and clinical medicine.

## Supplementary Material

btaf172_Supplementary_Data

## Data Availability

The DLPFC is available on the spatialLIBD website (http://research.libd.org/spatialLIBD/). The human breast cancer is available on the 10× Genomics website (https://support.10xgenomics.com/spatial-gene-expression/datasets). The mouse brain is available on the 10× Genomics website (https://www.10xgenomics.com/resources/datasets). The processed mouse olfactory bulb is available on the website (https://github.com/JinmiaoChenLab/SEDR_analyses/).

## Code availability

Source code is available at Github (https://github.com/Fuyh0628/STMGAMF) and Zenodo (https://zenodo.org/records/15103358).
