# Supplementary material for: A multi-view graph convolutional network framework based on adaptive adjacency matrix and multi-strategy fusion mechanism for identifying spatial domains
Source: Bioinformatics. 2025 Apr 15;41(4):btaf172. doi: 10.1093/bioinformatics/btaf172 (PMC12041416; doi:10.1093/bioinformatics/btaf172)
Supplement: btaf172_Supplementary_Data [file btaf172_supplementary_data.pdf]

# Supplementary Information for: A Multi-View Graph Convolutional Network Framework Based on Adaptive Adjacency Matrix and Multi-Strategy Fusion Mechanism for Identifying Spatial Domains

Yuhan Fu[1], Mengdi Nan[1], Qing Ren[1], Xiang Chen[1], Jie Gao[1,*]

## Contents

1. Methods
2. Supplementary Figures
3. Supplementary Results
4. Parameter Setting
5. Ablation Study
6. Stability Study
7. Significance Study
8. Evaluation Metrics
9. Datasets

[*]**Address:** School of Science, Jiangnan University, Wuxi, Jiangsu, 214122, China.
[†]**Corresponding Author:** Address correspondence to Jie Gao at the School of Science, Jiangnan University, Wuxi, Jiangsu, 214122, China. E-mail: gaojie@jiangnan.edu.cn

# 1 Methods

## 1.1 Data preprocessing

STMGAMF takes gene expression and spatial location information from ST data as its input. To reduce bias from technical or background noise in the ST data, all datasets are initially filtered to exclude spatial areas outside the main tissue areas. The raw gene expression typically contains many low-expression or low-variation genes, and we use the SCANPY (Wolf et al., 2018) toolkit to genetically filter and select features from the data, identifying the top 3,000 highly variation genes (Zeng et al., 2023). Finally, they are normalized using a scale factor. The normalization function is:

$$expression_{ij} = \frac{count_{ij}}{\sum_j count_{ij}} \times 10000. \tag{1}$$

## 1.2 Graph construction

To integrate gene expression and spatial location information effectively, we employ various adjacency graph construction methods based on spatial proximity and gene expression similarity. First, spatial proximity is assessed by analyzing the locations of adjacent spots within the tissue. Euclidean distance is an effective way to measure the physical distance between two spots in a multidimensional space. It is particularly suitable for calculating distances based on the spatial coordinates of the spots, as it captures the relationships between nearby spots within the organizational structure and accurately reflects the actual spatial arrangement of the organization (Turau, 1991; Bentley et al., 1977). Based on this, we construct a spatial adjacency graph $G_s = (A_s, X)$, where $A_s \in R^{N \times N}$ is the spatial adjacency matrix of $N$ spots and $X \in R^{N \times M}$ denotes the normalized gene expression matrix, where $M$ is the number of filtered genes. If both spot $i$ and $j$ are within a predefined radius $r$, then we set $A^s_{ij} = A^s_{ji} = 1$, and 0 otherwise (for the DLPFC, $r = 560$, while for the other datasets $r = 15$). This can be formalized as:

$$A^s_{ij} = \begin{cases} 1, & if\, d(i,j) \leq r \\ 0, & otherwise \end{cases}, \tag{2}$$

where $d(i,j)$ is the Euclidean distance between spot $i$ and $j$. The spatial adjacency graph accurately represents the tissue's spatial structure and ensures that the model reflects direct proximity based on physical location within the tissue. The graph takes into account only the spatial proximity of cells, but it does not effectively represent the biological significance of variations in gene expression.

To overcome the limitations of the spatial adjacency graph, we construct a feature adjacency graph based on gene expression similarity. This graph identifies cellular areas within a tissue that have similar functions or states by assessing the similarity of gene expression vectors, even if these areas are physically far apart. Specifically, we construct the k-nearest neighbor (KNN) (Cover and Hart, 1967) feature adjacency graph $G_f = (A_f, X)$, where $A_f \in R^{N \times N}$ is the feature adjacency matrix of $N$ spots. The edge weights of the feature adjacency matrix are calculated based on the cosine similarity. If spot $j$ is a neighbor of spot $i$, then we set $A^f_{ij} = 1$, and 0

otherwise ($k = 15$ by default). Cosine similarity is calculated as follows:

$$sim\left(x_i, x_j\right) = \frac{x_i \cdot x_j}{\left|x_i\right|\left|x_j\right|}. \tag{3}$$

The feature adjacency graph effectively identifies cells or areas with similar gene expression patterns. However, it may sometimes mistakenly group spatially distant spots in adjacent areas, overlooking the spatial structure. By building both a spatial adjacency graph and a feature adjacency graph, we enable the model to leverage spatial proximity and gene expression similarity simultaneously. This comprehensive approach effectively captures the complex relationships within the tissue.

### 1.3 Adaptive adjacency matrix construction

Traditional static adjacency matrices are usually created with predefined relationships. However, this approach may struggle to accurately depict the complex spatial structure of an organization or sample. In contrast, the adaptive adjacency matrix is designed to optimize relationships between spots by dynamically adjusting to changes in the input data. Here, we construct the adaptive adjacency matrix $A^{adapt}$ using both spatial adjacency and feature adjacency between the spots. The specific form is:

$$A_s^{adapt} = SparseMatrix\left(indices_s, values_s\right) \tag{4}$$

$$A_f^{adapt} = SparseMatrix\left(indices_f, values_f\right), \tag{5}$$

where $indices$ is the position index of non-zero elements in the adjacency matrix, indicating which spots are connected to each other; $values$ is the weight of edges in the adjacency matrix. The essence of the adaptive adjacency matrix lies in the dynamic learning of edge weights. For the spatial adjacency matrix $A_s$ and the feature adjacency matrix $A_f$, the indices of their non-zero elements are denoted as $I_s$ and $I_f$. Consequently, the initialized edge weights of the adaptive adjacency matrix are as follows:

$$A_{ij}^{adapt} = \begin{cases} \omega_{ij}^{s}, & if\ (i,j) \in I_s \\ \omega_{ij}^{f}, & if\ (i,j) \in I_f \\ 0, & otherwise \end{cases}, \tag{6}$$

where $\omega_{ij}^{s}$ and $\omega_{ij}^{f}$ are the weights of the edges based on the spatial adjacency matrix $A_s$ and the feature adjacency matrix $A_f$; $(i,j) \in I_s$ denotes that spot $i$ and $j$ are connected by spatial adjacency, while $(i,j) \in I_f$ denotes that spot $i$ and $j$ are connected by feature adjacency. During the training process of the model, the weights of the edges $\omega_{ij}$ can be updated through the gradient descent algorithm (Ruder, 2016).

By combining the prior information from the static adjacency matrix with a dynamic learning mechanism, the adaptive adjacency matrix not only captures spatial proximity and gene expression similarity but also adjusts flexibly to the complexity of the data.

### 1.4 Multi-view graph convolutional network encoder

In this model, we develop a multi-view learning framework based on a GCN (Kipf and Welling, 2016). The goal is to fully integrate the spatial and feature information within the graph structure while capturing the dependencies and interrelationships between various spots. By constructing multiple adjacency matrices and applying convolution operations to each one individually, the framework can extract specific information from different perspectives, thus combining the structural information of the graph with the feature data associated with the spots. Through multiple layers of convolution operations, the spatial information is gradually mined from local to global levels, enhancing identification accuracy in the spatial domains. The framework consists of three main modules: the spatial convolution module, the feature convolution module, and the joint convolution module, each describe in detail below.

#### 1.4.1 Spatial convolution

The purpose of the spatial convolution module is to capture the spatial information of the graph structure through the spatial adaptive adjacency matrix. Performing convolution operations on the spatial adaptive adjacency matrix $A_s^{adapt}$ allows for identifying and capturing spatial correlations between spots. The multilayer spatial convolution network applies the following hierarchical propagation rules:

$$H_s^{(l+1)} = \mathrm{ReLU}\left(A_s^{adapt}\tilde{D}_s^{-\frac{1}{2}}\tilde{A}_s\tilde{D}_s^{-\frac{1}{2}}H_s^{(l)}W_s^{(l)}\right), \tag{7}$$

where $W_s^{(l)}$ is the weight matrix of the $l$-th layer in the spatial convolution; the initial $H_s^{(0)} = X$; ReLU is the activation function; $\tilde{A}_s = A_s + I$, and $\tilde{D}_s$ is the diagonal degree matrix of $\tilde{A}_s$, with the diagonal element $\tilde{D}_{ii}^s = \sum_{j=1}^{N}\tilde{A}_{ij}^s$. This process ensures the spatial consistency of the graph data and the robustness of the propagation during the convolution process.

#### 1.4.2 Feature convolution

The feature convolution module is similar to the spatial convolution module, where we perform the convolution operation on the feature adaptive adjacency matrix $A_f^{adapt}$. The multilayer feature convolution network applies the following hierarchical propagation rules:

$$H_f^{(l+1)} = \mathrm{ReLU}\left(A_f^{adapt}\tilde{D}_f^{-\frac{1}{2}}\tilde{A}_f\tilde{D}_f^{-\frac{1}{2}}H_f^{(l)}W_f^{(l)}\right), \tag{8}$$

where $W_f^{(l)}$ is the weight matrix of the $l$-th layer in the feature convolution, the initial $H_f^{(0)} = X$; $\tilde{A}_f = A_f + I$, and $\tilde{D}_f$ is the diagonal degree matrix of $\tilde{A}_f$, with the diagonal element $\tilde{D}_{ii}^f = \sum_{j=1}^{N}\tilde{A}_{ij}^f$. This process focuses on effectively integrating spot features through graph convolution, enhancing the correlation of each feature within the graph structure.

#### 1.4.3 Joint convolution

In graph data, there is often a potential correlation between spatial information and feature information. In order to fully utilize the common information between the two, we design a

joint convolution module. The module performs convolution operations on the spatial adjacency matrix $A_s$ and feature adjacency matrix $A_f$ respectively, to extract the common information. The propagation rules are defined as follows:

$$H_{sj}^{(l+1)} = \text{ReLU}\left(\tilde{D}_s^{-\frac{1}{2}} \tilde{A}_s \tilde{D}_s^{-\frac{1}{2}} H_{sj}^{(l)} W_j^{(l)}\right) \tag{9}$$

$$H_{fj}^{(l+1)} = \text{ReLU}\left(\tilde{D}_f^{-\frac{1}{2}} \tilde{A}_f \tilde{D}_f^{-\frac{1}{2}} H_{fj}^{(l)} W_j^{(l)}\right), \tag{10}$$

where $W_j^{(l)}$ is the weight matrix of the $l$-th layer in the joint convolution, and the initial $H_{sj}^{(0)} = H_{fj}^{(0)} = X$. Joint convolution shares parameters between the spatial graph and the feature graph.

By using joint convolution, we can obtain the spatial joint embedding $H_{sj}$ and feature joint embedding $H_{fj}$, which can further define the joint embedding $H_j$ as:

$$H_j = \frac{H_{sj} + H_{fj}}{2}. \tag{11}$$

To improve the consistency of the representation between $H_{sj}$ and $H_{fj}$, we introduce consistency constraints defined as follows:

$$L_{con} = \left\| \tilde{H}_{sj} \tilde{H}_{sj}^T - \tilde{H}_{fj} \tilde{H}_{fj}^T \right\|_2^2, \tag{12}$$

where $\tilde{H}_{sj}$ and $\tilde{H}_{fj}$ are the normalization matrices of $H_{sj}$ and $H_{fj}$, respectively. This constraint promotes consistency between spatial embedding and feature embedding, enhancing the model's embedding quality.

## 1.5 Multi-strategy fusion

Different embedded features, such as gene expression, spatial information, and their combined representation, each contribute uniquely to the overall performance of the model in spatial domain identification tasks. To enhance identification accuracy, we design a multi-strategy fusion mechanism that adaptively integrates these features. The mechanism combines an attention-based (Tsotsos et al., 1995) fusion method and a fixed learnable parameter-based fusion method.

### 1.5.1 Fusion based on the attention mechanism

The attention mechanism highlights essential information while reducing irrelevant features by adjusting input feature weights adaptively, effectively capturing global dependencies. The model uses an attention mechanism to integrate various embeddings, including joint embedding, spatial embedding, and feature embeddings, allowing it to assess the significance of each embedding at different spatial locations. The specific process is as follows:

$$(a_j, a_s, a_f) = \text{att}(H_j, H_s, H_f), \tag{13}$$

where $a_j$, $a_s$ and $a_f$ are the attention coefficients of the joint embedding $H_j$, spatial embedding $H_s$ and feature embedding $H_f$, respectively. To obtain the attention coefficient $a_j$ for the joint embedding $H_j$, we first apply a linear transformation and then perform a dot multiplication with the shared attention vector $W_{sh}$:

$$a_j = \text{softmax}\left(W_{sh} \cdot \sigma\left(WH_j + b\right)\right), \tag{14}$$

where $\sigma$ is the tanh activation function, $W$ is the trainable weight matrix, $b$ is the bias vector. Similarly, we can compute the attention coefficients $a_s$ and $a_f$:

$$a_s = \text{softmax}\left(W_{sh} \cdot \sigma\left(WH_s + b\right)\right) \tag{15}$$

$$a_f = \text{softmax}\left(W_{sh} \cdot \sigma\left(WH_f + b\right)\right). \tag{16}$$

In this approach, all three embeddings use the same shared vector to compute the attention coefficients. This ensures a consistent attention mechanism is applied across the different embeddings. After calculating the attention coefficients for each embedding, we combine them, weighting each one according to its respective attention coefficient, to obtain the final attention fusion embedding $H_{attention}$:

$$H_{attention} = F\left(a_j \cdot H_j + a_s \cdot H_s + a_f \cdot H_f\right), \tag{17}$$

where $F$ is a single linear layer used to learn highly variable features of the underlying representation. This allows the model to adaptively adjust the weight of each embedding in the final fused representation, thereby enhancing the model's performance in spatial domain identification tasks.

### 1.5.2 Fusion based on fixed learnable weight parameters

Fixed learnable weight parameter-based fusion is a strategy for combining features through weighted summation and is particularly effective at capturing the overall relationships among different embedded features. In contrast to the attention mechanism, this approach emphasizes the stability of the fusion process while incorporating learnable weight parameters to adjust the weights of the embeddings dynamically. We introduce three learnable parameters, denoted $c_j$, $c_s$ and $c_f$, to dynamically adjust the weights of different embeddings. These parameters allow us to capture the interrelationships among the joint embedding $H_j$, spatial embedding $H_s$ and feature embedding $H_f$ to obtain a weighted combination of features. The details are as follows:

$$H_{learnable} = \tanh\left(c_j\right) \cdot H_j + \tanh\left(c_s\right) \cdot H_s + \tanh\left(c_f\right) \cdot H_f. \tag{18}$$

### 1.5.3 Final embedding

After computing both embeddings $H_{attention}$ and $H_{learnable}$, we fuse these two representations by averaging them to obtain the final embedding representation $H$:

$$H = \frac{H_{attention} + H_{learnable}}{2}. \tag{19}$$

This fusion merges the adaptive capability of the attention mechanism with the stability of fixed learnable weight parameters, enabling flexibility in capturing the significance of different embeddings while maintaining representation stability.

### 1.6 Zero-inflated negative binomial decoder

In scRNA-seq data, many genes are either not expressed in most cells or exhibit a large number of zero values due to technical factors. In order to better characterize the statistical properties of these data, the zero-inflated negative bino-mial distribution is considered a more appropriate model. This distribution can effectively deal with the overdispersion and zero-inflated problems present in the data and extract low-dimensional features from them. Additionally, ST data exhibits gene expression characteristics that are similar to those of scRNA-seq data, such as dispersion, variance that exceeds the mean, and a very high level of sparsity. In order to address the issues of zero inflation and missing data in ST data, the Zero-Inflated Negative Binomial (ZINB) (Yu et al., 2022) decoder structure is employed. This structure helps reconstruct the gene expression matrix and captures the complex global information inherent in ST data. ZINB is defined as follows:

$$f_{ZINB}\left(x|\pi,\mu,\theta\right) = \pi\delta_0\left(x\right) + \left(1-\pi\right)f_{NB}\left(x|\mu,\theta\right) \tag{20}$$

$$f_{NB}\left(x|\mu,\theta\right) = \frac{\Gamma\left(x+\theta\right)}{\Gamma\left(x+1\right)\Gamma\left(\theta\right)}\left(\frac{\theta}{\theta+\mu}\right)^{\theta}\left(\frac{\mu}{\theta+\mu}\right)^{x}, \tag{21}$$

where $\mu$ denotes the mean; $\theta$ denotes the dispersion; $\pi$ denotes the probability of a zero value; $\delta_0$ denotes an indicator function that $\delta_0 = 1$ when $x = 0$ and 0 otherwise. Three fully connected layers are used to estimate the parameters $\{\pi, \mu, \theta\}$ in the embedding representation $H$ as follows:

$$\Pi = \text{sigmoid}\left(W_\pi f_D\left(H\right)\right) \tag{22}$$

$$M = \exp\left(W_\mu f_D\left(H\right)\right) \tag{23}$$

$$\Theta = \exp\left(W_\theta f_D\left(H\right)\right), \tag{24}$$

where $f_D$ is a three-layer fully connected neural network; $W_\pi$, $W_\mu$, $W_\theta$ denote the learnable parameters of the network; $\Pi$, $M$, and $\Theta$ are all parameter matrices that indicate the probability of information loss, the mean of the network output, and the dispersion of the network output, respectively.

The negative log-likelihood of ZINB serves as the reconstruction loss for the original gene expression, which can be formulated as:

$$\begin{aligned} L_{ZINB} = -\sum_{i=1}^{N}\Bigg(&\delta_0(x_i)\log\left(\pi_i + (1-\pi_i)\left(\frac{\theta_i}{\theta_i+\mu_i}\right)^{\theta_i}\right) \\ &+ (1-\delta_0(x_i))\Big(\log(1-\pi_i) + \log f_{NB}(x_i|\mu_i,\theta_i)\Big)\Bigg) + \lambda\sum_{i=1}^{N}\pi_i^2, \end{aligned} \tag{25}$$

where the regularization term $\lambda\sum_{i=1}^{N}\pi_i^2$ is used to control the size of the zero-value probability

parameter $\pi$, which enhances the stability and generalization of the model.

## 1.7 Spatial regularization constraints

The spatial locations and gene expression patterns of cells or spatial spots within an organizational structure typically exhibit significant spatial dependence. Adjacent cells or spatial spots tend to show similar gene expression characteristics, suggesting they may belong to the same functional area. In contrast, more distant spots exhibit greater gene expression differences and may correspond to different areas (Stoltzfus et al., 2020). This spatial dependence highlights an important biological principle: spatially proximate spots are more relevant in terms of biological functions and tend to have more consistent gene expression patterns. To take full advantage of this spatial structural feature, we introduce spatial regularization constraints and use both similarity information and spatial adjacency information to calculate the regularization constraint loss. The spatial regularization constraint helps to define clear area boundaries and maintains spatial continuity in the potential space. It does this by reinforcing the similarity between adjacent spots while suppressing the similarity between non-adjacent spots, ensuring that the learned potential embeddings align with the actual spatial structure within the organization. Specifically, the spatial regularization constraint is based on the learned potential embeddings $H$. The similarity matrix $S$ is constructed based on the cosine similarity, which quantifies the degree of similarity between spatial spots. To enhance the similarity between adjacent spots, we define the adjacent spot loss as follows:

$$L_{nei} = \frac{1}{|G_{nei}|} \sum\nolimits_{(i,j)\in G_{nei}} \log \sigma\left(S_{ij}\right), \tag{26}$$

where $G_{nei}$ denotes the relationship between adjacent spots in the spatial regularization loss. $G_{ij}^{nei} = 1$, if spot $i$ and $j$ are adjacent, and 0 otherwise; $\sigma$ is the sigmoid activation function.

To reduce the similarity between non-adjacent spots, we define the non-adjacent spot loss as follows:

$$L_{neg} = \frac{1}{|G_{neg}|} \sum\nolimits_{(i,j)\in G_{neg}} \log\left(1 - \sigma\left(S_{ij}\right)\right), \tag{27}$$

where $G_{neg}$ denotes the relationship between non-adjacent spots in the spatial regularization loss. $G_{ij}^{neg} = 1$, if spot $i$ and $j$ are not adjacent, and 0 otherwise.

The final spatial regularization loss encompasses both the loss of adjacent spots and non-adjacent spots and is defined as:

$$L_{reg} = -\frac{1}{2}\left(\frac{1}{|G_{nei}|} \sum_{(i,j)\in G_{nei}} \log \sigma\left(S_{ij}\right) + \frac{1}{|G_{neg}|} \sum_{(i,j)\in G_{neg}} \log\left(1 - \sigma\left(S_{ij}\right)\right)\right). \tag{28}$$

By maximizing the similarity between adjacent spots and minimizing the similarity between non-adjacent spots, the model can create a spatial representation in potential space that aligns with the biological structure. This approach results in effective spatial partitioning and continuity.

### 1.8 Model loss function

During the model training process, we jointly optimize the multi-view GCN encoder, the ZINB decoder, and the spatial regularization constraints to enhance the model's performance in ST data analysis.

The final loss function of the model is defined as:

$$L = \alpha L_{ZINB} + \beta L_{con} + \gamma L_{reg}. \tag{29}$$

In this loss function, we set weight parameters $\alpha = 1$, $\beta = 10$, and $\gamma = 0.1$ to balance the effects of consistency loss, reconstruction loss, and spatial regularization loss. By appropriately adjusting these parameters to optimize the model, we can enhance its adaptability to the analysis needs of ST data.

# 2 Supplementary Figures

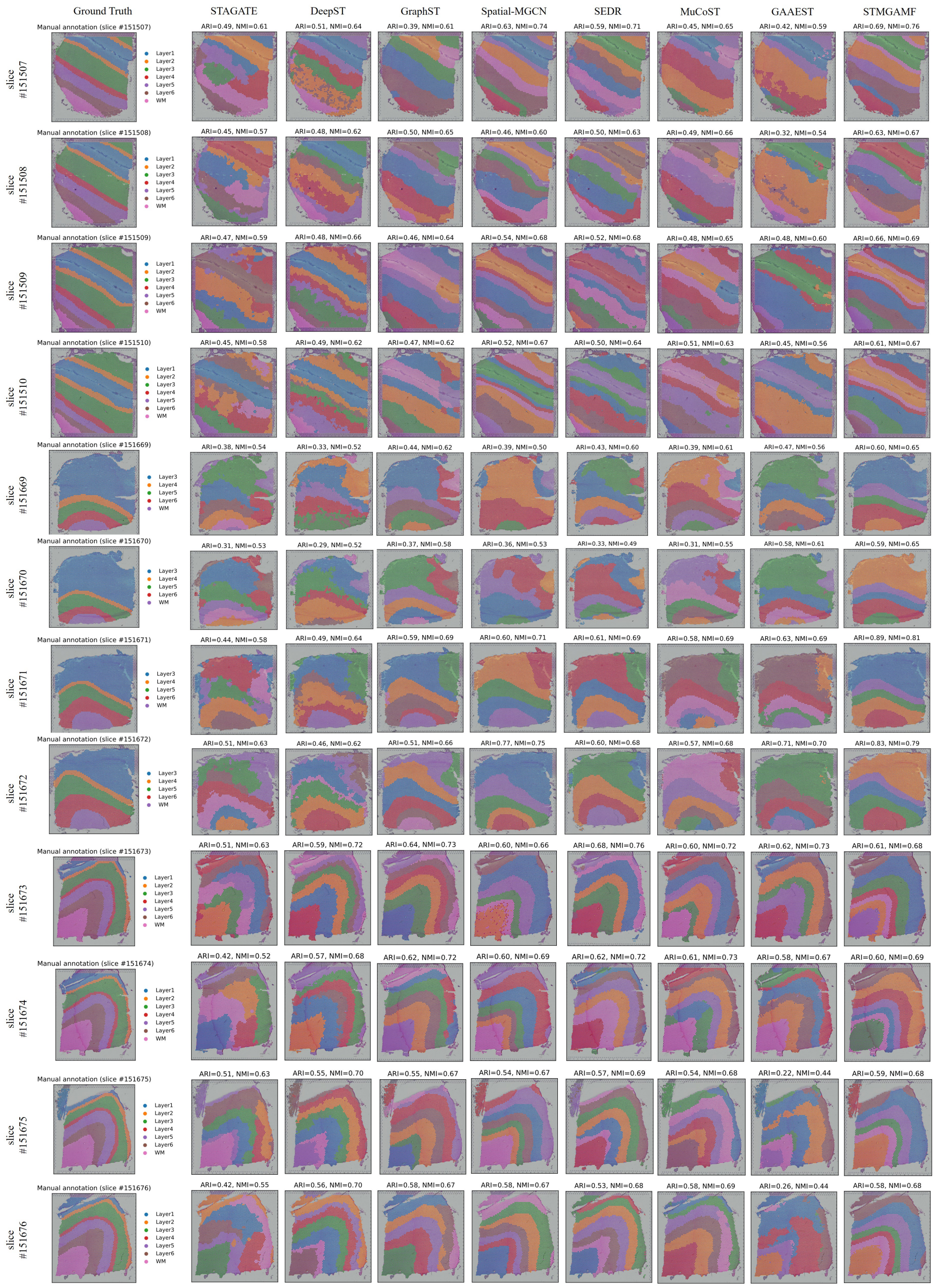


Figure S1: Manual annotations and comparison of spatial domains identified across 12 slices of DLPFC datasets from STMGAMF, STAGATE, DeepST, GraphST, Spatial-MGCN, SEDR, MuCoST and GAAEST.

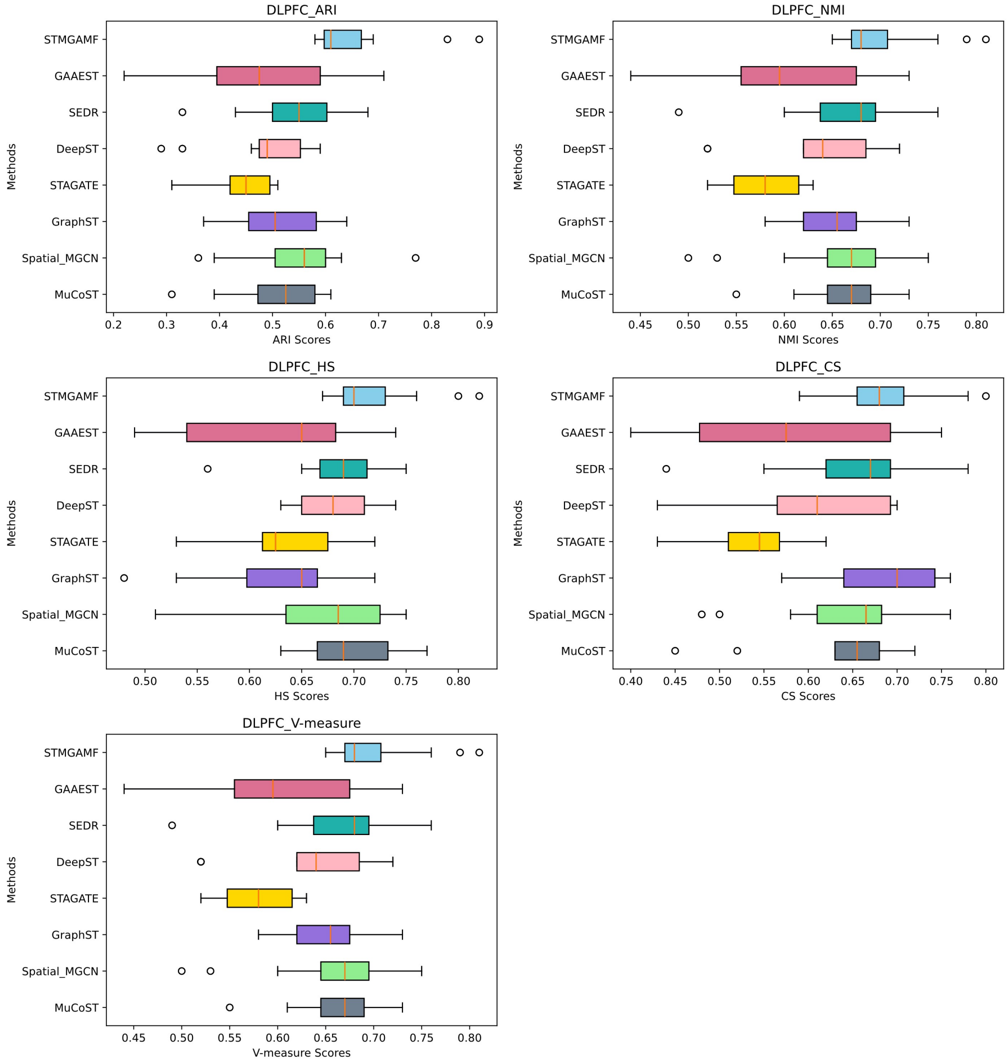


Figure S2: Comparison of evaluation criteria using ARI, NMI, HS, CS, and V-measure across 12 slices of DLPFC datasets from STMGAMF, STAGATE, DeepST, GraphST, Spatial-MGCN, SEDR, MuCoST and GAAEST.

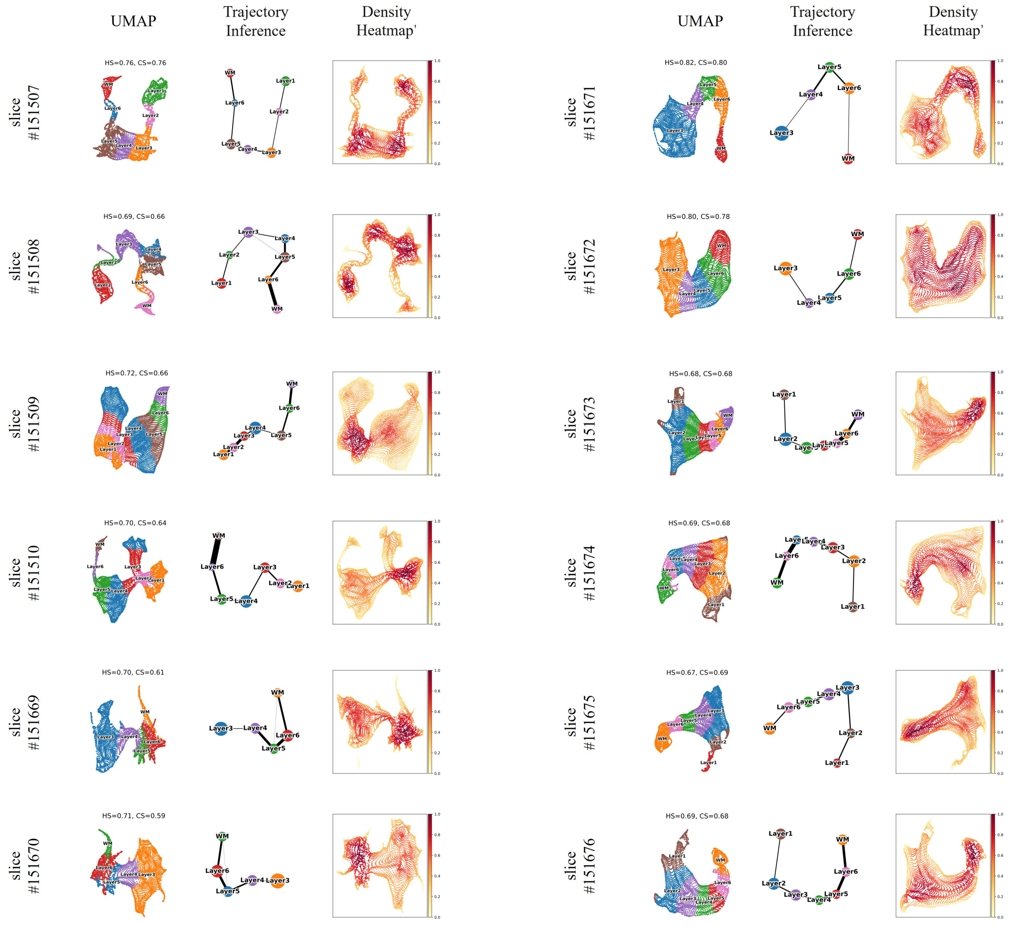


Figure S3: Results of STMGAMF for UMAP visualization, trajectory inference, and density heatmaps across 12 slices of DLPFC datasets.

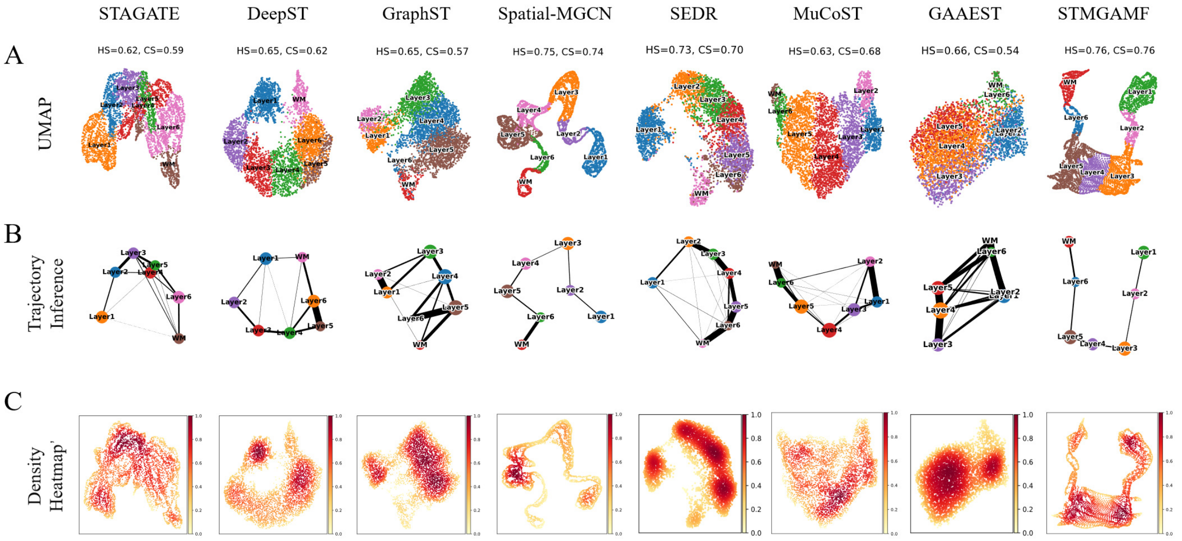


Figure S4: STMGAMF enhances the effectiveness of characterization learning when applied to DLPFC data. (A) UMAP visualization results of STMGAMF compared to seven baseline algorithms on slice #151507 in the DLPFC. (B) Trajectory inference results of STMGAMF compared to seven baseline algorithms on slice #151507 in the DLPFC. (C) The density heatmap results of STMGAMF compared to seven baseline algorithms on slice #151507 in the DLPFC.

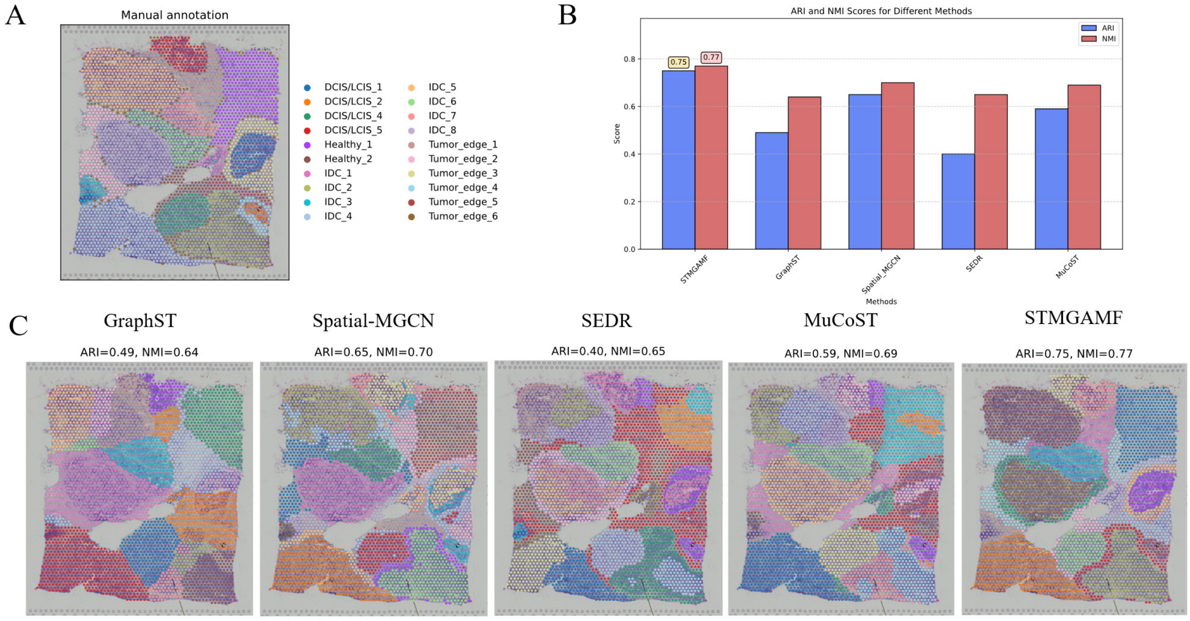


Figure S5: STMGAMF enhances the understanding of complex and heterogeneous human breast cancer tissue by focusing on its functional spatial domains. (A) Annotated sections of human breast cancer tissue samples. (B) Comparison of the ARI and NMI bar graphs between STMGAMF and four baseline algorithms applied to human breast cancer tissues. (C) Results of spatial domain identification from the STMGAMF and four baseline algorithms on human breast cancer.

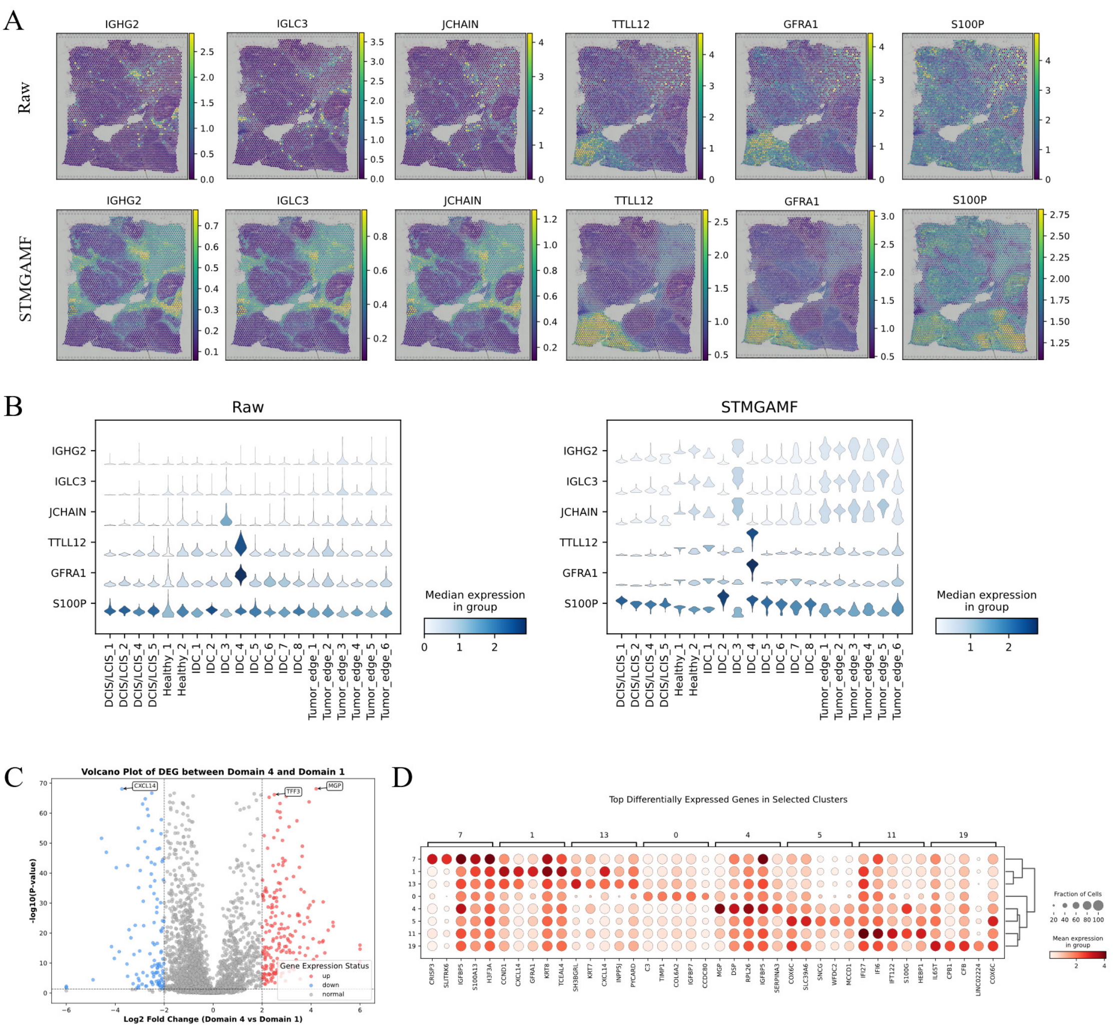


Figure S6: STMGAMF improves the interpretability of heterogeneous cancer tissues. (A) Visualization of raw and reconstructed expression of genes with spatial functions in the human breast cancer. (B) Violin plots comparing raw gene expression with the gene expression reconstructed by STMGAMF. (C) Analysis of differential expression between spatial domain 4 (DCIS/LCIS_1) and spatial domain 1 (IDC_4). Among the identified genes, MGP and TFF3 are up-regulated, while CXCL14 is down-regulated. (D) Expression heatmaps for the top five DEGs in spatial domains 4 (DCIS/LCIS_1), 11 (DCIS/LCIS_4), 0 (Healthy_1), 13 (IDC_1), 7 (IDC_2), 1 (IDC_4), 5 (IDC_5), and 19 (IDC_8).

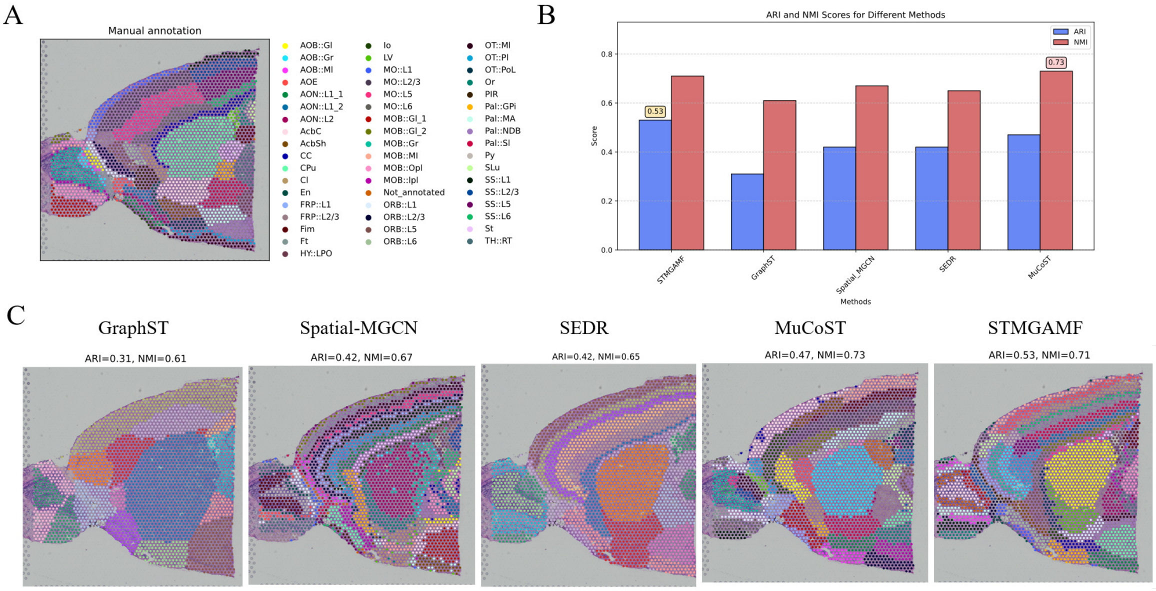


Figure S7: STMGAMF improves spatial domain identification in brain tissue. (A) Annotated sections of the anterior tissue of the mouse brain. (B) Comparison of the ARI and NMI bar graphs between STMGAMF and four baseline algorithms applied to the anterior tissue of the mouse brain. (C) Results of spatial domain identification from the STMGAMF and four baseline algorithms on the anterior tissue of the mouse brain.

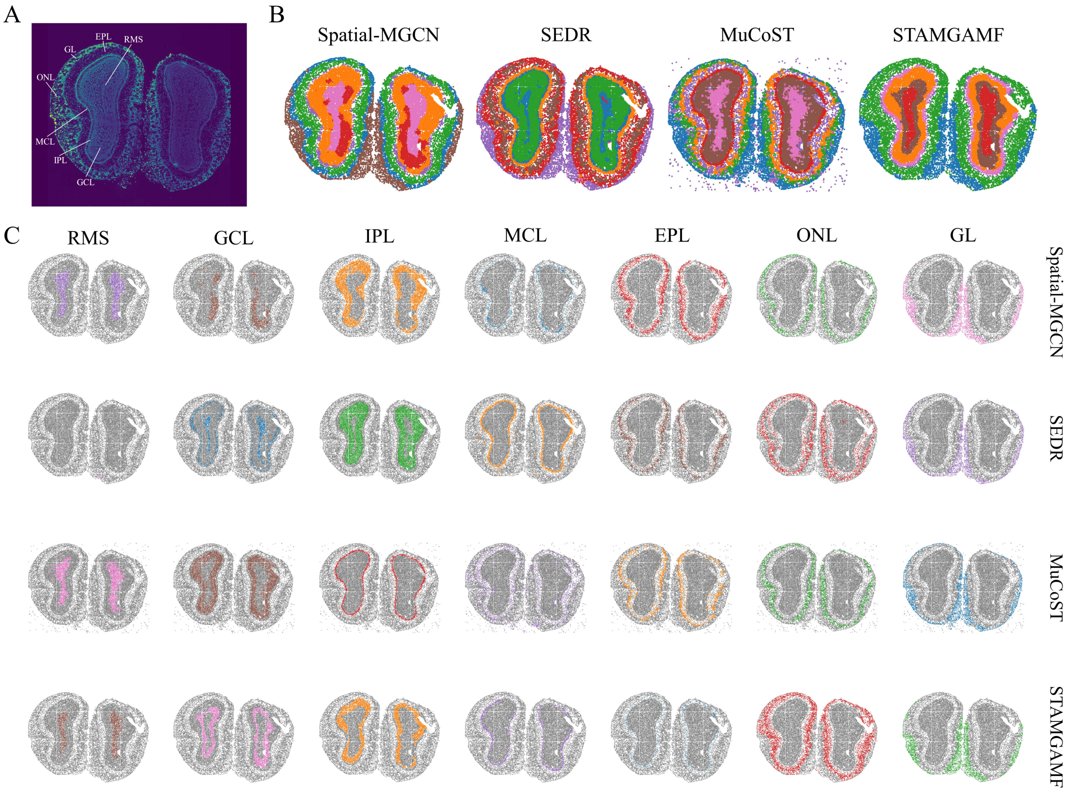


Figure S8: STMGAMF identifies the organizational structure of the mouse olfactory bulb obtained by the Stereo-seq platform. (A) The tissue annotation of DAPI-stained mouse olfactory bulb. (B) Results of spatial domain identification in the mouse olfactory bulb by STMGAMF and three baseline algorithms. (C) Visualization of the identified clusters in the mouse olfactory bulb for STMGAMF and the three baseline algorithms.

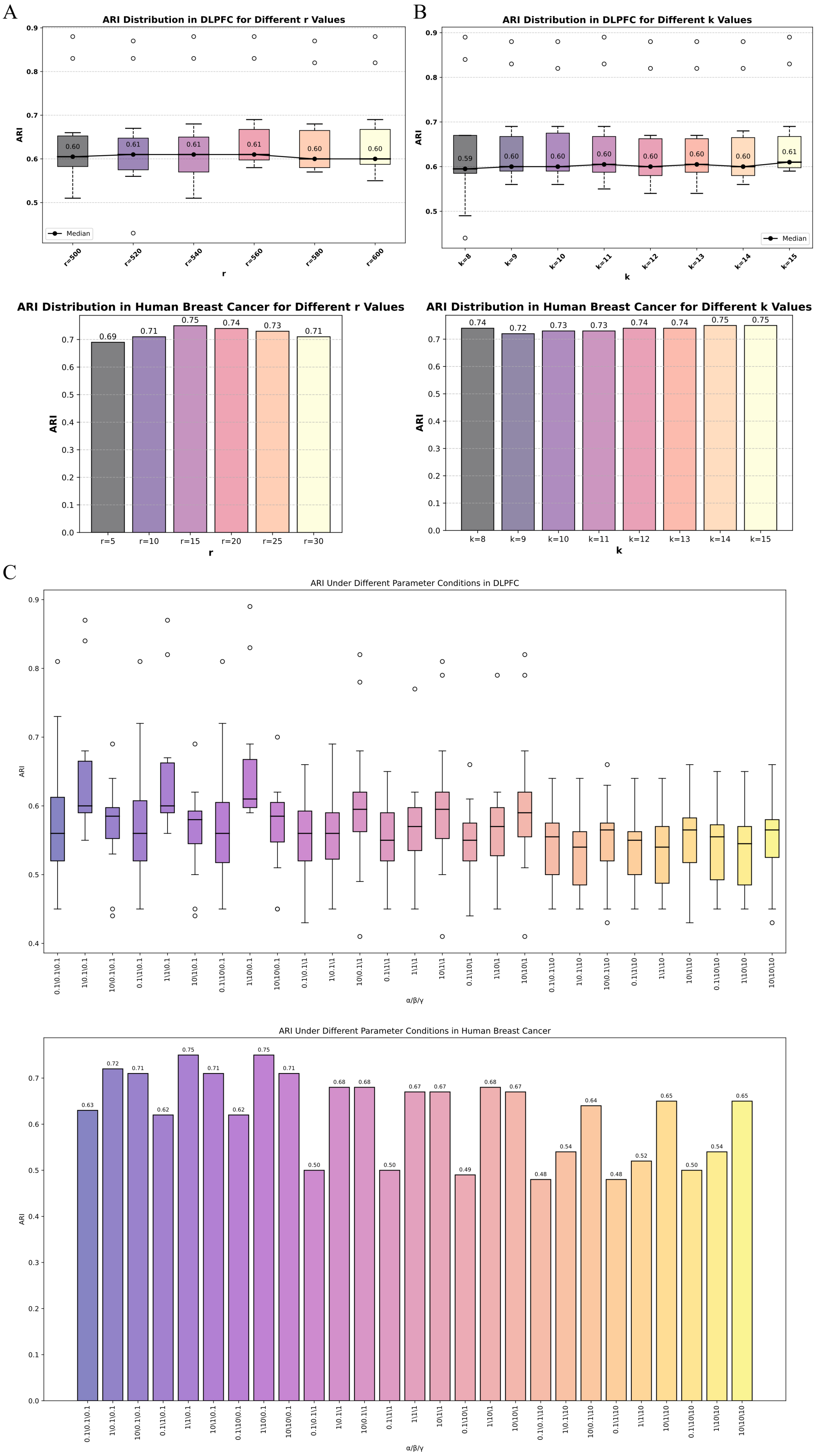


Figure S9: Parameter analysis. (A) Effects of different neighborhood radius $r$ configurations in the DLPFC and human breast cancer. (B) Effects of different nearest neighbor spot number $k$ configurations in the DLPFC and human breast cancer. (C) Effects of varying $\alpha$, $\beta$, and $\gamma$ combinations in the DLPFC and human breast cancer.

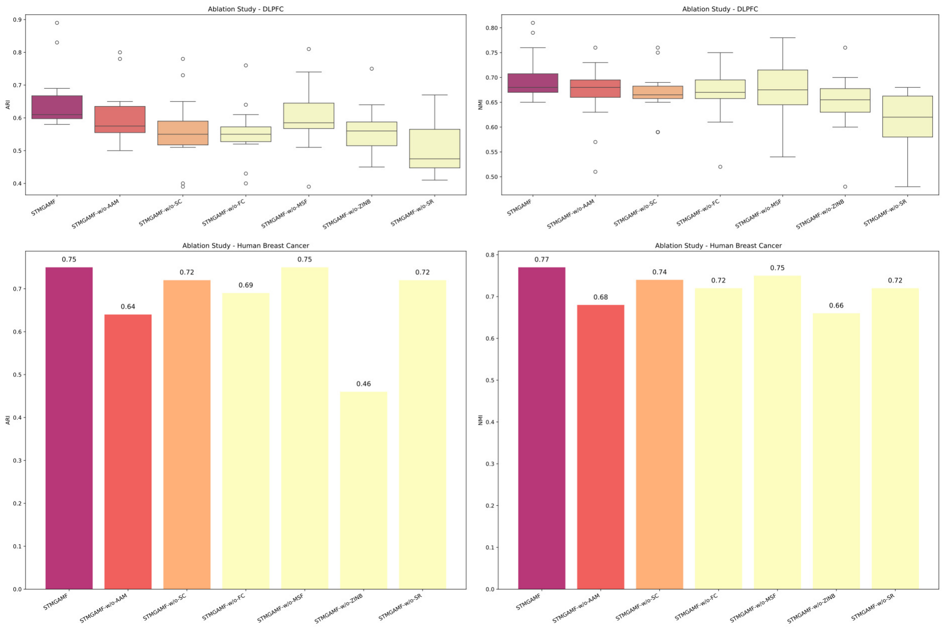


Figure S10: Boxplots of ARI and NMI of STMGAMF and its variants in the DLPFC and human breast cancer.

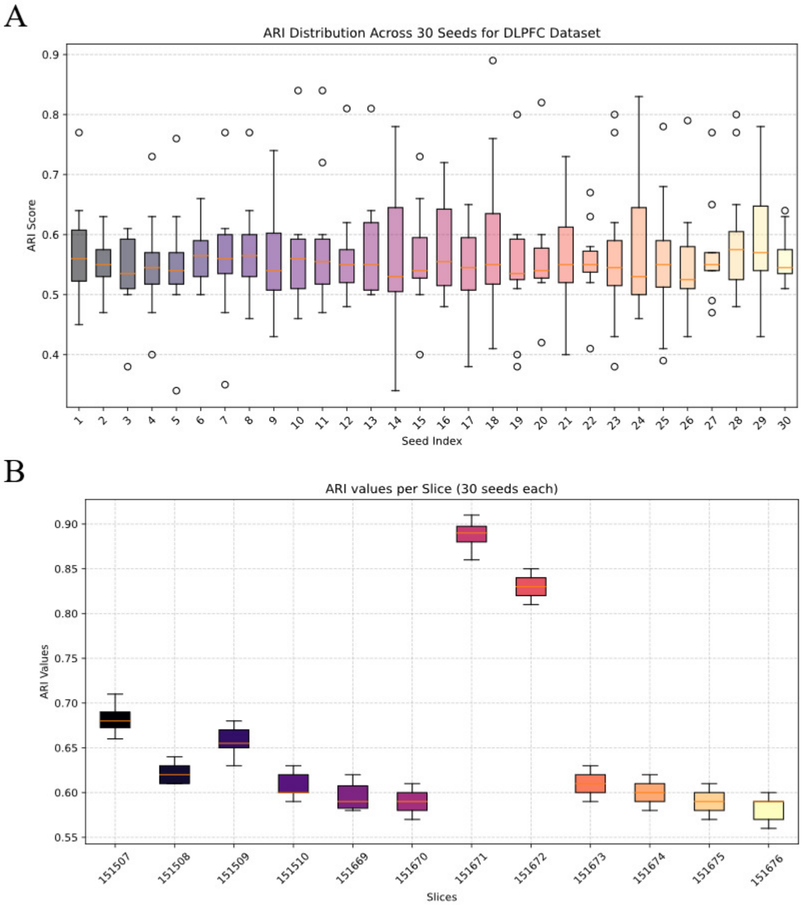


Figure S11: Stability analysis. (A) Effects of random seeds on STMGAMF in the DLPFC. (B) Repeated experiments of STMGAMF with fixed random seeds in the DLPFC.

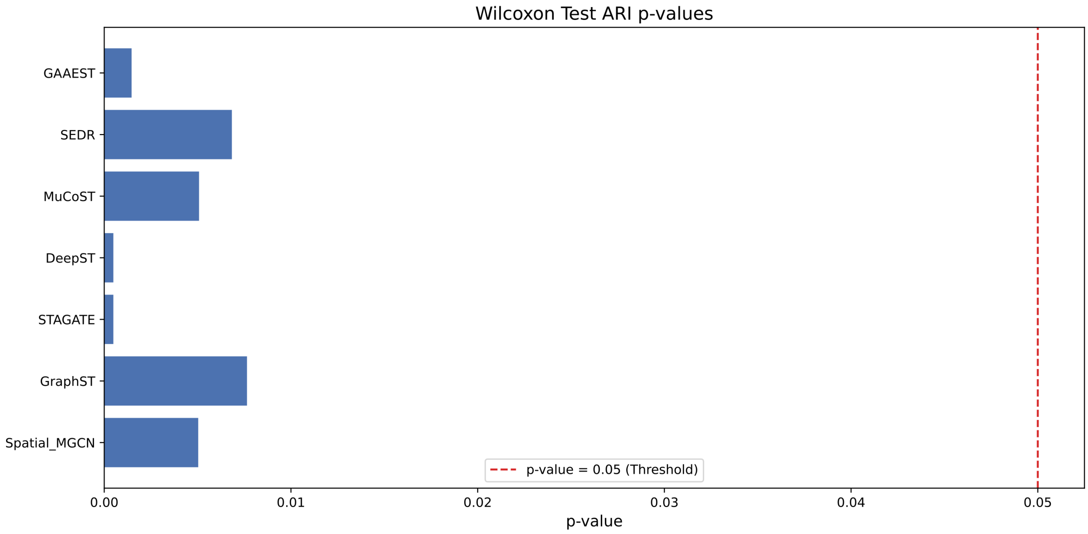


Figure S12: Comparison of ARI Using Wilcoxon Signed-Rank Test in the DLPFC.

# 3 Supplementary Results

## 3.1 STMGAMF demonstrates the spatial diversity of human breast cancer tissues and provides valuable biological insights

To validate the ability of STMGAMF to generalize within the complex spatial patterns and heterogeneous gene expression of cancer tissue, we test it on the 10× Visium dataset of human breast cancer tissues. The competing algorithms selected for this evaluation are GraphST, Spatial-MGCN, SEDR, and MuCoST, all of which achieve median ARI scores above 0.5 in the DLPFC. The human breast cancer is annotated into 20 spatial domains, classified into four main morphology types: Ductal Carcinoma In Situ/Lobular Carcinoma In Situ (DCIS/LCIS), Healthy, Invasive Ductal Carcinoma (IDC), and Tumor Edges (Xu et al., 2024) (Figure S5A).

In the spatial domain identification task, STMGAMF achieves the highest accuracy, with an ARI of 0.75 and an NMI of 0.77, significantly outperforming the other algorithms (Figure S5B). STMGAMF not only identifies tissue structures that are consistent with the manual annotations but also achieves the smoothest tissue boundaries with fewer outliers. Relative to other dispersed spatial domains, we identify eight significant spatial domains 4 (DCIS/LCIS_1), 11 (DCIS/LCIS_4), 0 (Healthy_1), 13 (IDC_1), 7 (IDC_2), 1 (IDC_4), 5 (IDC_5), and 19 (IDC_8) (Figure S5C). These domains conform to the manually annotated organizational structure and demonstrate clear boundaries, a notable advantage over other algorithms. Spatial-MGCN achieves the second-highest ARI of 0.65 and an NMI of 0.70. From the spatial domain identification result graph, it is observed that its identified spatial domains 0, 2, and 5 are mixed within DCIS/LCIS_1, while spatial domains 8 and 14 are contained in IDC_5. MuCoST achieves an ARI of 0.59 and an NMI of 0.69. The identified spatial domain 1 (IDC_4) is relatively close to the manual annotations, but spatial domains 9 and 2 were contained within Healthy_1, leading to fuzzy boundaries with no clear spatial patterns. GraphST achieves an ARI of 0.49 and an NMI of 0.64, and the areas identified are also heavily mixed. SEDR achieves an ARI of 0.40 and an NMI of 0.65, with confusion in some identified areas. Overall, these results indicate that STMGAMF produces more competitive spatial domain identification results, even in the context of heterogeneous human breast cancer tissues, successfully identifying significant spatial patterns and demonstrating impressive generalization ability.

We further validate the effectiveness of STMGAMF in enhancing or restoring spatial gene expression patterns by applying it to human breast cancer tissues. The experimental results demonstrate that certain genes with important spatial functions—such as IGHG2, IGLC3, JCHAIN, TTLL12, GFRA1, and S100P—exhibit smoother spatial expression and greater specificity in structural domains after undergoing denoising with STMGAMF (Figure S6A). Additionally, we compare the raw gene expression with the imputed gene expression using violin plots, which reveals significantly improved spatial expression patterns for these functionally important genes (Figure S6B). These findings suggest that STMGAMF effectively preserves the spatial distribution characteristics of key genes while eliminating noise, thereby greatly enhancing the interpretability of gene expression patterns.

We conduct a differential expression analysis between spatial domain 4 (DCIS/LCIS_1) and spatial domain 1 (IDC_4) to investigate the differences in gene expression between IDC and

DCIS/LCIS (Figure S6C). Our analysis identifies 314 significant differentially expressed genes (DEGs) between these two clusters. Further investigation suggests that the differential expression of these genes holds considerable biological significance. Among the upregulated genes in the upper right red area, Matrix Gla Protein (MGP) shows notable upregulation in the DCIS/LCIS area of breast cancer. This upregulation may have several biological implications, including maintaining the extracellular matrix environment, supporting tumor proliferation, promoting angiogenesis, and facilitating immune evasion by cancer cells. All these roles may contribute to a favorable growth environment for tumor cells during the non-invasive stages of breast cancer (Chen et al., 2023). MGP has also been shown to enhance current clinical biomarker assays for early cancer diagnosis (Du et al., 2022). Additionally, the upregulation of Trefoil Factor 3 (TFF3) is generally associated with increased tumor invasiveness, metastatic potential, and resistance to apoptosis. TFF3 may drive breast cancer progression through various mechanisms, such as promoting cell migration and invasion, supporting the epithelial-mesenchymal transition (EMT) process, enhancing angiogenesis, resisting apoptosis, and activating estrogen signaling pathways. It has been demonstrated that TFF3 acts as a predictive marker of endocrine response in breast cancer, enhancing the response of breast cancer to estrogen and thus further driving the malignant progression of estrogen receptor-positive (ER+) (May and Westley, 2015). In contrast, among the downregulated genes found in the blue area on the upper left, the downregulation of C-X-C Motif Chemokine Ligand 14 (CXCL14) may lead to a reduction of immune cells at the tumor site. This decrease may weaken the host's anti-tumor immune response, allowing cancer cells to evade immune surveillance and promoting tumor growth and metastasis. CXCL14 has been associated with breast cancer prognosis and has been shown to promote cancer growth (Sjöberg et al., 2019). Based on the analysis of these genes in spatial domain 4 (DCIS/LCIS_1) and spatial domain 1 (IDC_4), we conclude that in the DCIS/LCIS_1 area, tumor cells exhibit a stronger proliferative ability. In the IDC_4 area, tumor cells show greater invasiveness and metastatic potential.

To analyze the heterogeneity of cancer tissues, we compare the expression of the top five DEGs across various spatial domains: 4 (DCIS/LCIS_1), 11 (DCIS/LCIS_4), 0 (Healthy_1), 13 (IDC_1), 7 (IDC_2), 1 (IDC_4), 5 (IDC_5), and 19 (IDC_8) (Figure S6D). Our findings reveal significant heterogeneity among these clusters, indicating that different areas within the tumor exhibit distinct characteristics in terms of their biological functions.

The results indicate that STMGAMF is capable of distinguishing heterogeneous areas within tumors and revealing different biological functions specific to various spatial domains in tumor tissues. Additionally, it offers comprehensive biological insights into potential heterogeneity by integrating denoised data with differential expression analysis. This combination provides a crucial foundation for further research on the mechanisms behind breast cancer development and progression, as well as new perspectives for developing precision medicine strategies.

### 3.2 STMGAMF accurately identifies tissue structures in the mouse brain

Here, we test the spatial domain identification capabilities of STMGAMF, SEDR, GraphST, Spatial-MGCN, and MuCoST on the 10× Visium dataset of mouse anterior brain tissues to characterize fine-structure organizational architecture and region-specific functional genes com-

prehensively. Anterior mouse brain tissue slice is annotated into 52 spatial domains (Figure S7A).

In quantitatively evaluating the spatial domain identification task, STMGAMF achieves the highest ARI of 0.53 and a sub-optimal NMI of 0.71 (Figure S7B) while demonstrating a spatial domain hierarchy with smooth boundaries (Figure S7C). MuCoST obtains the second best ARI of 0.47 and the best NMI of 0.73; although its overall performance is superior, it shows localized speckled mixing in some subtle regions. Spatial-MGCN produces fuzzy identification results, making distinguishing the boundaries between domains difficult, achieving an ARI of 0.42 and an NMI of 0.67. Similarly, SEDR performs comparably to Spatial-MGCN, with the same ARI of 0.42 and an NMI of 0.65, but it still exhibits noticeable domain structure errors. GraphST struggles significantly with spatial domain identification, resulting in an ARI of 0.31 and an NMI of 0.61. The experimental results show that STMGAMF learns more informative and discriminative potential representations by deeply mining the intrinsic association between gene expression patterns and spatial location information through the multi-view GCN encoder. This approach improves the identification accuracy of spatial domains and demonstrates significant advantages in organizational structure resolution and functional gene region specificity analysis.

## 3.3 STMGAMF identifies ST tissue structures at different spatial resolutions

To further validate the applicability of STMGAMF in high-resolution ST datasets, we evaluate its spatial domain identification performance on the coronal mouse olfactory bulb tissue obtained by Stereo-seq. For comparison, we choose the best baseline algorithms on three datasets from the 10× Visium platform: Spatial-MGCN, SEDR, and MuCoST. Coronary mouse olfactory bulb tissue obtained by Stereo-seq contains seven anatomical domains (Xu et al., 2024): Olfactory Nerve Layer (ONL), Glomerular Layer (GL), External Plexiform Layer (EPL), Mitral Cell Layer (MCL), Internal Plexiform Layer (IPL), Granule Cell Layer (GCL) and Rostral Migratory Stream (RMS) (Figure S8A).

The experimental results show that STMGAMF accurately identify the laminar organization of the mouse olfactory bulb and match known tissue annotations (Figure S8B). Regarding outer layer tissues, ONL, GL, and EPL are successfully distinguished by Spatial-MGCN and STMGAMF. In contrast, SEDR and MuCoST fail to effectively distinguish GL and EPL, resulting in the mixing of the two. In terms of inner-layer organization, SEDR misclassifies some outer-layer patches as RMS. MuCoST can not identify the hierarchical structure among RMS, GCL, and IPL, and there is a more obvious mixing between clusters. In addition, Spatial-MGCN also fails to distinguish between RMS and GCL; in contrast, STMGAMF is the only algorithm that could accurately identify all inner tissues (Figure S8C). This result further validates the advantages of the adaptive adjacency matrix in complex data environments. STMGAMF can dynamically adjust the weights according to the data changes to more accurately adapt to the complexity of the organization structure and achieve better spatial domain identification performance.

# 4 Parameter Setting

The learning rate is set to 1e-3, which effectively controls the step size for parameter updates, ensuring stable convergence while mitigating oscillations during training. We apply a weight decay of 5e-4 and adopt the Adam optimizer for optimization. For constructing spatial and feature graphs, the neighborhood radius r and the number of nearest neighbors k are dynamically adjusted based on the dataset scale and spatial resolution characteristics. Expressly, $k$ is set to a default value of 15 to ensure the constructed topological graph effectively captures spatial proximity relationships among nodes. For the DLPFC, we set $r = 560$, while for all other datasets, $r = 15$, allowing for the effective extraction of local spatial structures (Figure S9A, Figure S9B). Regarding the weighting parameters $\alpha$, $\beta$, and $\gamma$, we conduct a systematic sensitivity analysis across the DLPFC and human breast cancer within the search space $\{0.1, 1, 10\}$. The experimental results demonstrate that the model achieves optimal performance in benchmark tests when $\alpha = 1$, $\beta = 10$, and $\gamma = 0.1$ (Figure S9C). Therefore, this configuration is finalized as the standard setting for our model.

## 5 Ablation Study

We systematically remove the adaptive adjacency matrix, the multi-view GCN encoder, the multi-strategy fusion mechanism, the ZINB decoder, and the spatial regularization constraints to assess their independent contribution to model performance.

* STMGAMF-w/o-AAM: The adaptive adjacency matrix is removed;
* STMGAMF-w/o-SC: The direct spatial convolution and joint convolution operations in the multi-view GCN encoder are removed, and only the direct feature convolution is retained;
* STMGAMF-w/o-FC: The direct feature convolution and joint convolution operations in the multi-view GCN encoder are removed, and only the direct spatial convolution is retained;
* STMGAMF-w/o-MSF: The multi-strategy fusion mechanism is removed;
* STMGAMF-w/o-ZINB: The ZINB decoder is removed, and a fully connected layer replaces the decoder and is not included in the total loss;
* STMGAMF-w/o-SR: The spatial regularization constraint is removed, and the component is not included in the total loss.

The experimental results are shown in Figure S10. STMGAMF consistently performs superiorly in all variants. Specifically, STMGAMF-w/o-SR shows the most significant performance degradation in the DLPFC, with the median ARI and NMI dropping to 0.48 and 0.62, respectively, which demonstrates the critical role of spatial regularization constraints in preserving the spatial structural features and maintaining the spatial continuity effectively. Compared with STMGAMF-w/o-MSF, both median ARI and median NMI scores of STMGAMF in the DLPFC are significantly higher, which further validates the effectiveness of the multi-strategy fusion mechanism to flexibly capture the importance of different embeddings, thus enhancing the embedding quality and safeguarding the model performance. For the human breast cancer, STMGAMF-w/o-ZINB has the lowest ARI and NMI scores of 0.46 and 0.66, respectively, demonstrating the importance of the ZINB decoder in coping with the high-noise and high sparsity characteristics of ST data, which can effectively capture the global probability distribution of the data. On this dataset, the ARI and NMI scores of STMGAMF-w/o-AAM are the second lowest, 0.64 and 0.68, respectively, indicating that the adaptive adjacency matrix can flexibly adjust the weights according to the complexity of the data to capture the complexity of the spatial structure better. In addition, STMGAMF-w/o-SC and STMGAMF-w/o-FC show mediocre performance on both datasets, suggesting that the multi-view GCN encoder effectively extracts and integrates the information of gene expression and spatial location in different viewpoints, which improves the clustering performance. Overall, the ablation experiments highlight the indispensability of the adaptive adjacency matrix, multi-view GCN encoder, multi-strategy fusion mechanism, ZINB decoder, and spatial regularization constraints in STMGAMF. The synergy of these components enables STMGAMF to identify spatial domain features more accurately and significantly improve model performance.

## 6 Stability Study

Several experimental verifications have been performed to explore the stability of STMGAMF. First, we conduct a random seed experiment. We train STMGAMF independently under multiple random seeds (1-30) and calculate the ARI scores in the DLPFC (Figure S11A). The results show good stability, and the mean and median ARI of the twelve slices fluctuate between 0.54 and 0.60. Secondly, we conduct repeated experiments (Figure S11B). With the random seed of each slice fixed, we repeat the experiment 30 times for each slice and observe that the floating range of the ARI value of each slice is about $\pm 0.02$, which further verifies the stability of the model.

# 7 Significance Study

we have conducted Friedman and Wilcoxon signed-rank tests on the DLPFC dataset's ARI scores to assess the statistical significance of STMGAMF. For the Friedman test, we obtain $ARI = 39.84$ and $p_valu e = 1.35E{-}06$ (the p-value is much less than 0.05, indicating a significant difference). The Friedman test results show statistically significant differences between the compared algorithms, which suggests that the performance of different approaches is statistically distinguishable. In addition, the Wilcoxon signed rank test results show that STMGAMF outperforms all baseline algorithms with p-values below 0.05 (Figure S12). This indicates that the improvement of STMGAMF is statistically reliable and not a fluctuating result due to chance.

# 8 Evaluation Metrics

We use several metrics to evaluate the quality of clustering results, including the Adjusted Rand Index (ARI), Normalized Mutual Information (NMI), Homogeneity Score (HS), Completeness Score (CS), and V-measure. These metrics assess the performance of clustering results from various perspectives and effectively reflect how well the model performs in spatial domain identification tasks.

## 8.1 Adjusted Rand Index

The Adjusted Rand Index (ARI) enhances the classical Rand Index (RI) by accounting for the influence of random clustering. By adjusting for the expected value of random assignments, the ARI offers a more reliable and robust measure of clustering accuracy compared to unadjusted metrics:

$$ARI = \frac{\sum_{ij}\binom{n_{ij}}{2} - \left(\sum_i \binom{a_i}{2} \sum_j \binom{b_j}{2}\right) \Big/ \binom{n}{2}}{\frac{1}{2}\left(\sum_i \binom{a_i}{2} + \sum_j \binom{b_j}{2}\right) - \left(\sum_i \binom{a_i}{2} \sum_j \binom{b_j}{2}\right) \Big/ \binom{n}{2}}. \tag{30}$$

The value of ARI ranges between $[-1, 1]$. An ARI score of 1 signifies a perfect match, indicating that the clustering results are identical to the ground truth. A score near 0 suggests that the clustering results are no better than random assignments. Conversely, a negative ARI value indicates that the clustering results deviate from the true labels to a degree worse than random chance.

## 8.2 Normalized Mutual Information

The Normalized Mutual Information (NMI) is an information-theoretic metric used to evaluate the agreement between true and predicted labels in clustering. By normalizing Mutual Information (MI), NMI accounts for the number of label categories, ensuring reliable and consistent comparisons across datasets:

$$NMI\left(U, V\right) = \frac{2 \cdot I\left(U, V\right)}{H\left(U\right) + H\left(V\right)}. \tag{31}$$

The value of NMI ranges between $[0, 1]$. An NMI score of 1 indicates a perfect match between the clustering results and the true labels, while an NMI score of 0 signifies complete independence between the two.

## 8.3 Homogeneity Score

The Homogeneity Score (HS) evaluates the extent to which each cluster in a clustering result comprises samples (data spots) from the same true category:

$$HS = 1 - \frac{H\left(V|U\right)}{H\left(V\right)}. \tag{32}$$

HS takes values between $[0, 1]$. An HS score of 1 indicates complete homogeneity, meaning each cluster contains samples from only one category. Conversely, an HS score of 0 signifies low homogeneity, where samples from multiple categories are mixed within the cluster.

## 8.4 Completeness Score

The Completeness Score (CS) evaluates how effectively the clustering algorithm assigns all samples of the same category to a single cluster:

$$CS = 1 - \frac{H\left(U|V\right)}{H\left(U\right)}. \tag{33}$$

CS takes values between $[0, 1]$. A CS score of 1 indicates complete completeness, meaning all samples from each category are assigned to the same cluster. Conversely, a CS score of 0 signifies low completeness, indicating that samples from each category are widely distributed across multiple clusters.

## 8.5 V-measure

The V-measure is a metric used to evaluate the quality of clustering algorithms. It combines two main components: homogeneity and completeness, into a single score:

$$V - measure = \frac{(1+\eta) \cdot HS \cdot CS}{\eta \cdot HS + CS}. \tag{34}$$

The V-measure takes values between $[0, 1]$. A V-measure score of 1 indicates perfect clustering, signifying that both homogeneity and completeness are high. On the other hand, a V-measure score of 0 indicates a complete failure to accurately reflect the true category structure of the data.

# 9 Datasets

To systematically evaluate the performance of STMGAMF, we conduct comprehensive benchmarking experiments across four representative publicly available ST datasets. The first dataset is LIBD human dorsolateral prefrontal cortex (DLPFC), encompassing six distinct cortical layers. The second dataset is human breast cancer, representing heterogeneous tumor microenvironments. The third dataset is derived from the anterior tissue of the mouse brain, and all three of these datasets are generated using the 10$\times$ Visium platform. To validate the applicability of STMGAMF in high-resolution ST datasets, we also include a high-resolution Stereo-seq dataset of mouse olfactory bulb tissue.

## References


Bentley, J. L., Stanat, D. F., and Williams Jr, E. H. (1977). The complexity of finding fixed-radius near neighbors. *Information processing letters*, 6(6):209–212.

Chen, Y., Jiang, Y., Cui, T., Hou, L., Zhao, R., Bo, S., Zou, L., and Yin, C. (2023). Creatine ameliorates high-fat diet-induced obesity by regulation of lipolysis and lipophagy in brown adipose tissue and liver. *Biochimie*, 209:85–94.

Cover, T. and Hart, P. (1967). Nearest neighbor pattern classification. *IEEE transactions on information theory*, 13(1):21–27.

Du, T., Pan, L., Zheng, C., Chen, K., Yang, Y., Chen, J., Chao, X., Li, M., Lu, J., Luo, R., et al. (2022). Matrix gla protein (mgp), gata3, and trps1: a novel diagnostic panel to determine breast origin. *Breast cancer research*, 24(1):70.

Kipf, T. N. and Welling, M. (2016). Semi-supervised classification with graph convolutional networks. *arXiv preprint arXiv:1609.02907*.

May, F. E. and Westley, B. R. (2015). Tff3 is a valuable predictive biomarker of endocrine response in metastatic breast cancer. *Endocrine-related cancer*, 22(3):465.

Ruder, S. (2016). An overview of gradient descent optimization algorithms. *arXiv preprint arXiv:1609.04747*.

Sjöberg, E., Meyrath, M., Milde, L., Herrera, M., Lövrot, J., Hägerstrand, D., Frings, O., Bartish, M., Rolny, C., Sonnhammer, E., et al. (2019). A novel ackr2-dependent role of fibroblast-derived cxcl14 in epithelial-to-mesenchymal transition and metastasis of breast cancer. *Clinical cancer research*, 25(12):3702–3717.

Stoltzfus, C. R., Filipek, J., Gern, B. H., Olin, B. E., Leal, J. M., Wu, Y., Lyons-Cohen, M. R., Huang, J. Y., Paz-Stoltzfus, C. L., Plumlee, C. R., et al. (2020). Cytomap: a spatial analysis toolbox reveals features of myeloid cell organization in lymphoid tissues. *Cell reports*, 31(3).

Tsotsos, J. K., Culhane, S. M., Wai, W. Y. K., Lai, Y., Davis, N., and Nuflo, F. (1995). Modeling visual attention via selective tuning. *Artificial intelligence*, 78(1-2):507–545.

Turau, V. (1991). Fixed-radius near neighbors search. *Information processing letters*, 39(4):201–203.

Wolf, F. A., Angerer, P., and Theis, F. J. (2018). Scanpy: large-scale single-cell gene expression data analysis. *Genome biology*, 19:1–5.

Xu, H., Fu, H., Long, Y., Ang, K. S., Sethi, R., Chong, K., Li, M., Uddamvathanak, R., Lee, H. K., Ling, J., et al. (2024). Unsupervised spatially embedded deep representation of spatial transcriptomics. *Genome Medicine*, 16(1):12.

Yu, Z., Lu, Y., Wang, Y., Tang, F., Wong, K.-C., and Li, X. (2022). Zinb-based graph embedding autoencoder for single-cell rna-seq interpretations. In *Proceedings of the AAAI conference on artificial intelligence*, volume 36, pages 4671–4679.

Zeng, Y., Yin, R., Luo, M., Chen, J., Pan, Z., Lu, Y., Yu, W., and Yang, Y. (2023). Identifying spatial domain by adapting transcriptomics with histology through contrastive learning. *Briefings in Bioinformatics*, 24(2):bbad048.
